# Development, Testing, Parameterization, and Calibration of a Human Physiologically Based Pharmacokinetic Model for the Plasticizer, Hexamoll^®^ Diisononyl-Cyclohexane-1, 2-Dicarboxylate Using *In Silico*, *In Vitro*, and Human Biomonitoring Data

**DOI:** 10.3389/fphar.2019.01394

**Published:** 2019-11-29

**Authors:** Kevin McNally, Craig Sams, George Loizou

**Affiliations:** Exposure and Health Consequences, Health and Safety Executive, Buxton, United Kingdom

**Keywords:** plasticizer, Hexamoll®, PBPK, *in silico*, *in vitro*, human, biomonitoring

## Abstract

A physiologically based pharmacokinetic model for Hexamoll^®^ diisononyl-cyclohexane-1, 2-dicarboxylate was developed to interpret the biokinetics in humans after single oral doses. The model was parameterized with *in vitro* and *in silico* derived parameters and uncertainty and sensitivity analysis was used during the model development process to assess structure, biological plausibility and behavior prior to simulation and analysis of human biological monitoring data. The model provided good simulations of the urinary excretion (Curine) of two metabolites; cyclohexane-1,2-dicarboxylic acid mono hydroxyisononyl ester (OH-MINCH) and cyclohexane-1, 2-dicarboxylic acid mono carboxyisononyl ester (cx-MINCH) from the biotransformation of mono-isononyl-cyclohexane-1, 2-dicarboxylate (MINCH), the monoester metabolite of di-isononyl-cyclohexane-1,2-dicarboxylate. However, good simulations could be obtained, with and without, a lymphatic compartment. Selection of an appropriate model structure was informed by sensitivity analysis which could identify and quantify the contribution to variability in Curine by parameters, such as, the fraction of oral dose that directly entered the lymphatic compartment and therefore by-passed the liver and the fraction of MINCH bio-transformed to cx-MINCH and OH-MINCH. By constraining these parameters within biologically plausible limits the presence of a lymphatic compartment was deemed an important component of model structure. Furthermore, the use of sensitivity analysis is important in the evaluation of uncertainty around *in silico* derived parameters. By quantifying their impact on model output sufficient confidence in the use of a model should be afforded. This type of approach could expand the use of physiologically based pharmacokinetic models since parameterization with *in silico* techniques allows for rapid model development. This in turn could assist in reducing the use of animals in toxicological evaluations by enhancing the utility of “read across” techniques.

## Introduction

Plasticizers from different classes of chemicals are used in the manufacture of plastics. They are used to promote plasticity, that is, the ability to be shaped and molded, to increase flexibility and reduce brittleness. The most commonly used plasticizers are the phthalates, a term which describes dialkyl- or alkylarylesters of 1,2-benzenedicarboxylic acid. The length of the ester chain determines the industrial application, with alkyl chain lengths from three to 13 carbons widely used in polymers such as polyvinyl chloride (PVC). For health effects evaluation phthalates have commonly been grouped as low molecular weight (three to six carbon alkyl chain length) and high molecular weight (HMW; seven or more carbon alkyl chain). The plasticizer content of soft PVC, for example, can reach up to 40% (Koch and Calafat, 2009). Products containing plasticizers include floorings, roofings, wall coverings and cables, clothing, packaging materials and toys (David et al., 2001; Koch and Calafat, 2009). However, phthalates can leach into the surrounding media because they are physically, not chemically, bound to the polymer. Therefore, plasticizers can enter the environment and directly enter the human body. Environmental exposure may encompass many exposure sources, but data from fasting humans suggest real world exposure to HMW phthalate plasticizers is likely predominantly attributable to dietary exposures, whereas low molecular weight phthalates (potential in a non-plasticizer application) attributable to use of personal care products (Koch et al., 2013a).

In a review by Koch and Calafat (2009) certain phthalates like e.g. di-(2-ethylhexyl) phthalate (DEHP), di-iso-nonylphthalate, butylbenzyl phthalate, di-iso-butyl phthalate, di-*n*-butyl phthalate, and dipentyl phthalate can modulate the endogenous production of foetal testicular testosterone and insulin-like factor 3 in rats (Gray and Gangolli, 1986; Gray Jr et al., 2000; Borch et al., 2006; Howdeshell et al., 2007; Furr et al., 2014) . These phthalates have also been grouped as suspected human endocrine disrupters (Koch and Calafat, 2009), though there is variation in their potential to cause developmental effects in rats that suggests the HMW phthalates do not warrant hazard classification (ECHA, 2018). The ubiquitous presence of plasticizers in the environment, in humans and the demonstration of toxicity in animal studies for some specific phthalates has led to multiple hazard and safety assessments by regulatory agencies globally.

When available, human biomonitoring (HBM) can be an important part of the risk assessment (RA) and risk management of compounds such as the plasticizers. HBM is the repeated controlled measurement of a chemical, its metabolites, or biochemical markers in accessible samples such as bodily fluids (e.g., urine, blood, and saliva), exhaled air, and hair (Manno et al., 2010). In risk characterization, HBM is often superior to other methods of exposure assessment, such as personal air measurements or dermal deposition assessments, because actual estimated body burden or biologically effective dose is a composite measure of multiple routes of exposure, of the differences in individual behavior (e.g., personal hygiene), work rate (characterized by different respiration rates), physiology, metabolism, and hence susceptibility (Boogaard et al., 2011). Uncertainty in external exposure assessment due to inter- and intra-individual variability can also be reduced by using HBM if the measured biomarker, either parent chemical or metabolite, is proportionately related to the ultimate toxic entity (Boogaard et al., 2011). In general, urine is the matrix of choice for HBM of non-persistent chemicals such as plasticizers because urine is an easily accessible matrix which can be used also with children, women of child-bearing age and other sub-populations for quantification of these compounds and/or their metabolites, and it is commonly the most important quantitative route of excretion for chemicals.

The ability to estimate organ and tissue dose or “tissue dosimetry” from body burdens calculated using HBM should further improve the correlation of exposure to health effects (Loizou and Hogg, 2011). Physiologically based pharmacokinetic (PBPK) modeling is a powerful means of simulating the factors that determine tissue dose within any biological organism and consequently, it is correlation with health effects (Clewell and Andersen, 1985; Krishnan and Andersen, 1994; Rostami-Hodjegan and Tucker, 2007). The value of PBPK models is that they are tools for integrating *in vitro*, *in silico*, and *in vivo* mechanistic, pharmacokinetic, and toxicological information achieved through their explicit mathematical description of important anatomical, physiological and biochemical determinants of chemical uptake, distribution, and elimination. Thus, PBPK modeling is increasingly being used in chemical RA (Chiu et al., 2007; Loizou et al., 2008; WHO, 2010).

In this study we present a PBPK model developed to interpret the urinary excretion of metabolites in humans of di-isononyl-cyclohexane-1,2-dicarboxylate (Hexamoll^®^ DINCH), a HMW phthalate substitute, after a single oral dose (Koch et al., 2013b). The structure of the model, initially informed by previous studies with DEHP (Kessler et al., 2012), includes a sub- model for the first monoester metabolite, mono-isononyl-cyclohexane-1,2-dicarboxylate (MINCH) and the urinary excretion of the first two oxidized metabolites of MINCH, cyclohexane-1,2-dicarboxylic acid mono hydroxyisononyl ester (OH-MINCH) and cyclohexane-1,2-dicarboxylic acid mono carboxyisononyl ester (cx-MINCH). A simple description of the oral uptake of DINCH via the stomach and gut with a portion of the oral dose entering the hepatic portal vein and another portion into the lymphatic system via the lacteals is included (Kessler et al., 2012). The model was parameterized with *in vitro* and *in silico* methods, that is, measured intrinsic hepatic clearance scaled from *in vitro* to *in vivo* and predicted octanol–water partition coefficient (PC) (Log P_ow_) values which, in turn, were used to predict parameters such as plasma unbound fraction and tissue:blood PCs. Also, the sufficiency and relevance of PBPK model structure and the sensitivity of model output to *in vitro* and *in silico* derived model parameters was investigated using an approach based on global sensitivity analysis (GSA) as part of the ongoing development of a good PBPK modeling practice (Barton et al., 2007; Loizou et al., 2008; Barton et al., 2009; WHO, 2010; Paini et al., 2017; Clewell et al., 2019; Ellison et al., 2019; Fabian et al., 2019).

## Materials and Methods

### Experimental

#### Chemicals

Pooled human microsomes were purchased from (Tebu-bio, Peterborough, UK). The microsomes were prepared from a pool of 50 liver samples; mixed gender (20 mg protein ml^-1^). DINCH and MINCH were provided by BASF. All chemicals used were of analytical grade or higher.

#### Analysis

Samples were analysed by liquid chromatography (Shimadzu Prominence) with tandem mass spectrometry detection (AB Sciex API 3200) using electrospray ionization. Ion optics, temperatures and gas flows were optimized on our individual system. All analyses used a Synergi Hydro-RP column (150 × 2 mm; 4 µ; Phenomenex) in conjunction with a methanol: 20mM ammonium acetate (0.1% acetic acid) gradient. Sample injection volume was 2 µl.

##### In Vitro Incubations

**Figure 1:**
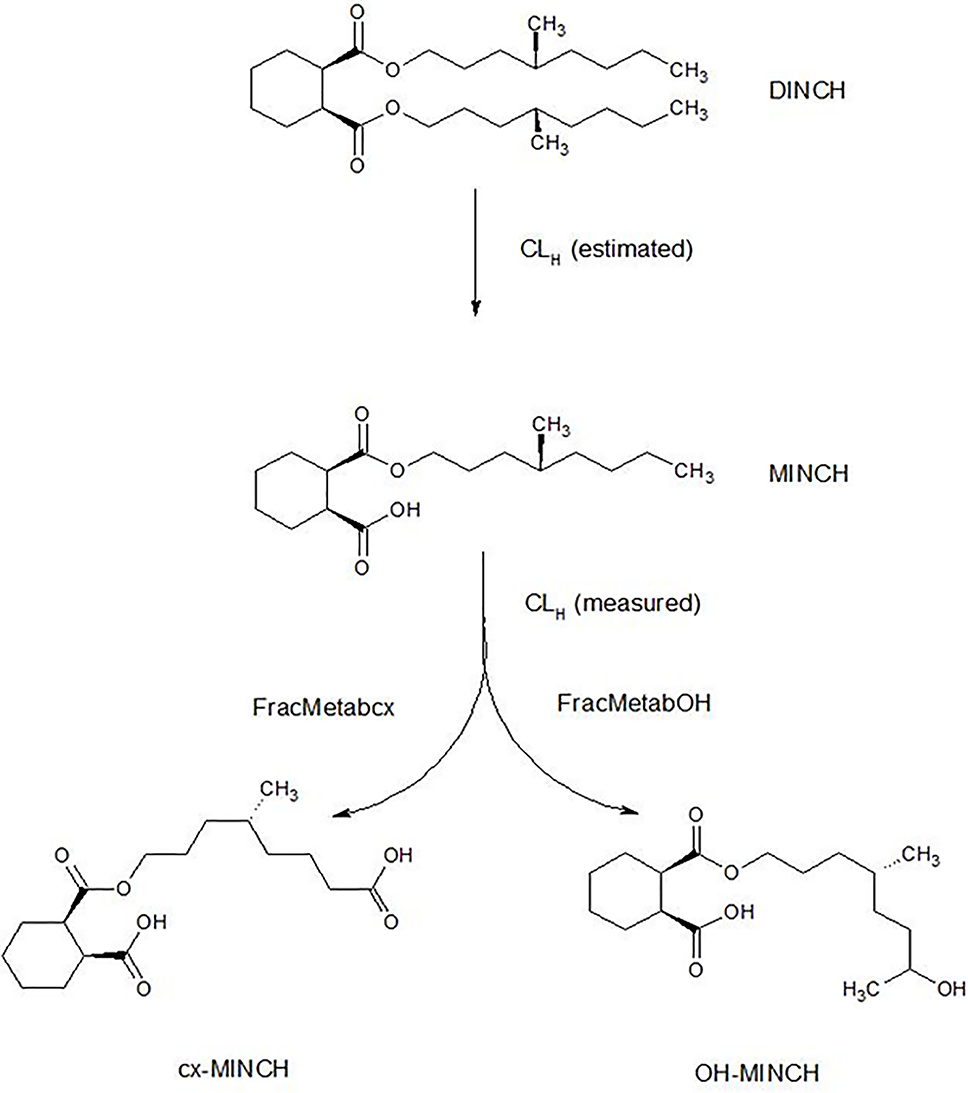
Postulated DINCH metabolism in humans showing only those metabolites measured in human biological monitoring and described in the PBPK model.

The very high lipophilicity of DINCH resulted in the formation of an insoluble film on the surface of the reaction medium and precluded the measurement of *in vitro* clearance. Therefore, the *in vitro* clearance of MINCH only was possible (**Figure 1**).

The NADPH regenerating system consisted of the following final concentrations: 1.3 mM NADP^+^; 3.3 mM glucose-6-phosphate; 5 mM magnesium chloride; 0.4 U/ml glucose-6-phosphate dehydrogenase; 50 mM phosphate buffer (pH 7.4). Final microsomal protein concentration was 0.5 mg/ml. Incubations were performed in polypropylene tubes and pre-warmed reaction mixtures were started by addition of substrate dissolved in acetonitrile. The final acetonitrile concentration was less than 1% and, typically, a substrate concentration of 10 µM was used (initial investigations were performed to check solubility in the reaction mixture). Incubations were conducted in a water bath at 37°C. At the time points chosen for measurement, tubes were mixed by inversion and an aliquot removed and quenched by adding to an equal volume of ice-cold methanol followed by centrifugation to precipitate the protein as a pellet. The supernatant was removed for analysis. Three replicates were sampled at each time point. Control incubations consisted of reaction mix excluding glucose-6-phosphate dehydrogenase (for evaluation of non-specific binding) and reaction mix excluding microsomes (for evaluation of substrate stability).

The method of Jones and Houston (2004) was used to determine the *in vitro* half-life of substrate depletion. At least three independent incubations were performed and results were assessed visually for reproducibility. However, due to differences in sampling time points between experiments, results from individual incubations were not combined.

##### Determination of In Vitro Intrinsic Clearance

**Figure 2:**
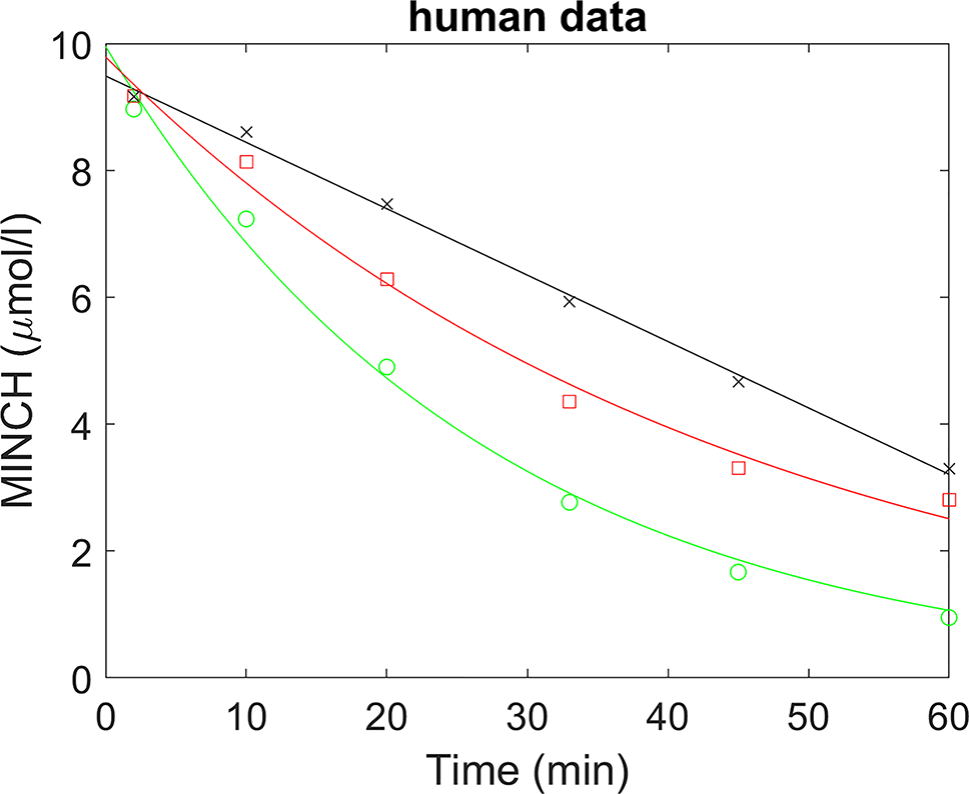
Determination of half-life for the estimation of *in vitro* intrinsic clearance of MINCH in human liver microsomes where, (×) represents non-specific binding, (○) total liver metabolism, and (□) specific liver metabolism.

A straight line was fitted to the non-specific binding data and a scaling factor calculated (the intercept divided by the fitted value) for each time point; thus multiplying the non-specific binding data by the scaling factors would generate scaled non-specific binding data that were approximately constant over all times (**Figure 2**). When applied to parent chemical (in this case, MINCH) concentration, this factor can account for losses such as non-specific binding to microsomes and the reaction vessel. The mean of the repeated measured concentrations of MINCH at each time point was multiplied by the corresponding scaling factor. An exponential decay function,

(1) $$y=be^{-t/k}$$

was fitted to the scaled data using a non-linear fit (the Levenberg–Marquardt nonlinear least squares algorithm) where, *y* is the scaled concentration, *b* is the fitted value at *t* = *0* and the decay constant is given by *k*.

*In vitro* clearance, *CL* *_in vitro_* (milliliter per minute per milligram microsomal protein) in human hepatic microsomes was calculated using the half-life (*T* *_½_*) derived from the decay constant (*k*) using the following equations (Obach et al., 1997):

(2) $$\mathrm{in vitro}T_{1/2}=\frac{\ln(2)}{k}$$

(3) $${\mathrm{CL}}_{\mathrm{in vitro}=\frac{\ln(2)}{\mathrm{in vitro}T_{1/2}}\times \frac{\mathrm{ml incubation}}{\mathrm{mg microsomes}}}$$

Where, *ml incubation* is the volume (milliliter) of the incubation medium and *mg microsomes* is the mass (milligram) of microsomes in the incubation medium.

The sensitivity of model output to the intrinsic clearance term was tested within the PBPK model structure as described in the section on sensitivity analysis. The half-life (T½) for MINCH accounted for approximately 1.5% of variability in urinary metabolite excretion during the first 2 h post-oral uptake of DINCH and almost zero for the remainder of the simulation period. Therefore, the contribution of residual metabolism in the absence of glucose-6-phosphate dehydrogenase to non-specific binding was not measured as this would have made an insignificant contribution to simulations.

#### Calculation of *In Vivo* Clearance

The intrinsic hepatic clearance CL_int_H_ was calculated using the following formula adapted from Obach (1999):

(4) $${\mathrm{CL}}_{{\mathrm{int}}_{H}}={\mathrm{CL}}_{\mathrm{in vitro}}\times \mathrm{MPY}\times \mathrm{Vli}\times 60$$

Where, *MPY* is the microsomal protein yield per g liver (milligram per gram), *Vli* is mass of the liver (gram) and the 60 converts from minutes to hours.

Whole liver plasma clearance *CL* *_H_* (liter per hour) was calculated assuming the well-stirred model of hepatic clearance taking into account the unbound fraction in plasma, *fu* and the red blood cells to plasma ratio, C_RBC_/C_P_, using the following equation (Yang et al., 2007):

(5) $${\mathrm{CL}}_{H}=\frac{Q_{H}\cdot \mathrm{fu}\cdot {\mathrm{CL}}_{\mathrm{int}\_H}}{Q_{H}+\mathrm{fu}\cdot CL_{\mathrm{int}\_H}/(C_{\mathrm{RBC}}/C_{p})}$$

Where, *Q* *_H_* (liter per hour) is the blood flow to the liver as a proportion of cardiac output.

The intrinsic gut clearance CL_int_gut_ was calculated similarly as described for hepatic clearance, but substituting *MPY* *_gut_* and *Vgu* for *MPY* and *Vli*, respectively, in Equation 3. The resulting calculated CL_int_gut_ was used in place of CL_int_H_ for calculation of CL_gut_.

#### Prediction of Log P_ow_

The common quantitative descriptor of lipophilicity, the octanol–water PC P_ow_, is defined as the ratio of the concentrations of a neutral compound in organic and aqueous phases of a two-compartment system under equilibrium conditions. It is mostly used in its logarithmic form, Log P_ow_.

A large number of different calculation methods have been derived for estimating the Log P_ow_ of chemical structures. A comparison of calculation methods identified the ACDLogP as one of the best performing methods (Mannhold et al., 2009).

The ACDLogP algorithm is based on contributions of separate atoms, structural fragments, and intramolecular interactions between different fragments. These contributions have been derived from an ACD/Labs^1^ internal database of over 18,400 structures with experimental Log P_ow_ values (Petrauskas and Kolovanov, 2000). Therefore, the ACDLogP method implemented in the ACD/ChemSketch 2014 software^2^ was used to calculate Log P_ow_ values required to then calculate the tissue:blood PCs for the plasticizer and it is primary metabolites (**Table 1**).

**Table 1:** Tissue:blood partition coefficients and plasma fraction unbound predicted using Log P_ow_.

	DINCH	MINCH
Log Po:w	9.77	5.17
Tissue:blood partition coefficient		
Adipose	49.77	29.10
Liver	5.89	54.80
Muscle	3.29	7.51
Blood cells	3.01	6.67
Gut	7.40	25.20
Spleen	3.70	12.20
*Stomach* *^1^ (gut)*	7.40	25.20
Rapidly Perfused (spleen)	3.70	12.20
Slowly Perfused (muscle)	3.29	7.51
Plasma Fraction Unbound	0.000125	0.014648

^1^Compartments in italics have surrogate values from another organ compartment. The corresponding surrogate organ compartment is in parentheses.

#### Prediction of Tissue: Blood Partition Coefficients

Two tissue-composition-based algorithms for the calculation of tissue:blood PCs were used in this study. The method of Poulin and Haddad (2012) which was developed for the prediction of the tissue distribution of highly lipophilic compounds, defined as chemicals with a Log P_ow_ > 5.8, was used for DINCH (**Table 1**). The method of Schmitt (2008) which was developed to predict the tissue distribution of chemicals with Log P_ow_ < 5.17 was used to predict the PCs of the monoester, MINCH (**Table 1**).

The algorithm of Poulin and Haddad (2012) was implemented as a Microsoft^®^ Excel Add-in whereas a modified version of the algorithm of Schmitt (2008) was available within the httk: R Package for High-Throughput Toxicokinetics (Pearce et al., 2017).

Where the tissue-composition-based algorithms did not provide a tissue:blood PC for a particular compartment the value from a surrogate organ or tissue was assumed. These are presented in italicized text with the surrogate organ or tissue in brackets **Table 1**.

#### Prediction of Plasma Fraction Unbound

In general, in the presence of plasma proteins the fraction of a compound unbound in plasma decreases as the lipophilicity of a compound increases (van de Waterbeemd et al., 2001; Peters, 2011). The fraction unbound (*fu*) was calculated from *log ((1-fu)/fu)* with the following equation:

(6) $$\mathrm{fu}=\frac{1}{10^{x}+1}$$

Where, *x* = 0.4485 *logP* − 0.4782

When *x* is the equation for the prediction of *fu* for a chemical with a predominantly uncharged state at pH 7.4 (Lobell and Sivarajah, 2003) (**Table 1**).

#### Calculation of Fraction Metabolized

The proportion of MINCH metabolized to cx- and OH-MINCH, represented by FracMetab (FracMetabcx to cx- and FracMetabOH to OH-MINCH) (**Table 2**) for each volunteer was estimated by expressing all the biological monitoring data (MINCH, OH-MINCH, cx-MINCH, cyclohexane-1,2-dicarboxylic acid, oxo-MINCH, and MCHxCH) in moles and dividing the amount of cx- and OH-MINCH each by the total sum of all metabolites (**Table 2**).

**Table 2:** Volunteer specific parameters.

	Volunteer		
	A	B	C
**Body weight (kg)**	89	90	82
Dose (mg kg^-1^)	0.558	0.552	0.606
Mean (sd) rate of urine production (L h^-1^)	0.104 (0.053)	0.158 (0.086)	0.190 (0.242)
Mean (sd )creatinine concentration (g L^-1^)	1.278 (0.605)	0.962 (0.672)	1.278 (0.831)
**Fraction Metabolised**			
FracMetab to cx_MINCH	0.045	0.048	0.049
			
FracMetab to OH_MINCH	0.250	0.238	0.202

#### Data

The biological monitoring data described in Koch et al. (2013b) were kindly provided by Dr. Rainer Otter of BASF, SE. Briefly, DINCH was administered orally to three healthy male volunteers, aged between 26 and 38 years, weighing between 82 and 90 kg. Approximately 50 mg DINCH dissolved in 0.25 ml of ethanol was applied in an edible chocolate coated waffle cup containing tea or coffee during breakfast. The resulting respective doses for the three individuals were between 0.552 and 0.606 mg kg^-1^ body weight (BW) (**Table 2**). The first urine sample was collected prior to dosage followed by consecutive sample collection up to 48 h post-dose.

### The Physiologically Based Pharmacokinetic Model

**Figure 3:**
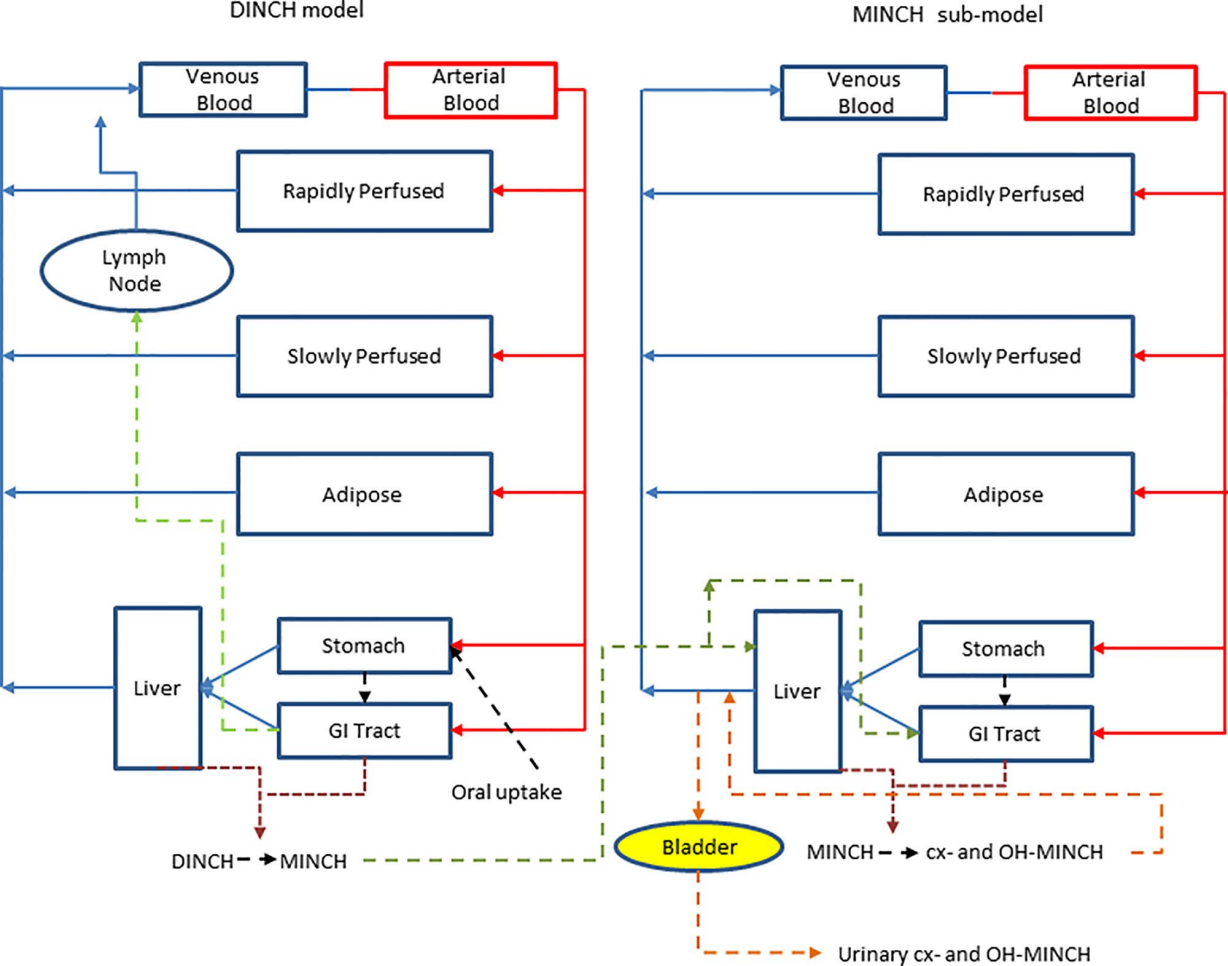
A schematic of the model for DINCH and sub-model for MINCH. The DINCH model contained a lymphatic compartment (– – -) which received a portion of the DINCH oral dose from the stomach and gut, which by-passes the liver and enters the venous blood via the lymph node represented by the blue arrow. The biotransformation of DINCH to MINCH occurs in both the liver and gut. In the MINCH sub-model the biotransformation of MINCH to cx- and OH-MINCH is ascribed only to the liver and urinary excretion of metabolites were described with first-order elimination rate constants and a bladder compartment.

A PBPK model was developed to study the metabolism of DINCH in humans after single oral doses. The model includes a bladder compartment to simulate fluctuations in metabolite concentration in the urine caused by micturition (Franks et al., 2006), a description of absorption from both the stomach and the gut (Loizou and Spendiff, 2004) and a simple model of the lymphatic system describing uptake of DINCH via the lacteals in the intestine and entering the venous blood after bypassing the liver (Kessler et al., 2012) (**Figure 3**). A lymphatic compartment was included as chemicals with Log Pow > 5 are potentially suitable for lymphatic transport through lacteals (Brocks and Davies, 2018).The dose that entered the lymphatic system via the gut, FracDOSE was coded in the model as a proportion of administered dose, with the complementary (greater) proportion entering the liver via the portal vein (**Table 3**). The model described the metabolism of DINCH to MINCH in the gut with both DINCH and MINCH entering the systemic circulation via uptake from the gut like DEHP to mono(2-ethylhexyl)phthalate (MEHP) (Kessler et al., 2012). Therefore, a sub-model was added to describe the kinetics of MINCH. The two models were connected at the level of the liver. The model for DINCH differed from the sub-model for MINCH only with the presence of a lymphatic compartment and the sub-model for MINCH having the bladder compartment; otherwise both models had a stomach and gut draining into the liver, an adipose, slowly and rapidly perfused and blood compartment (**Figure 3**).

**Table 3:** Physiological and kinetic default values used in PBPK model and probability distributions applied for uncertainty and sensitivity analyses.

Physiological Parameters	Abbreviation	Default Value	Distribution
			
**Body weight (kg)**	BW	72.3	N(72.3,9.05)
% BW			
Total vascularised tissues	VT	0.95	–
Liver	VLiC	3.09	N(3.09, 0.8)
Fat	VFaC	19.5	LN(3.42,0.43)
Gut	VGuC	1.50	U(1.19,1.84)
Stomach	VStC	0.22	N(0.22, 0.07)
Slowly perfused tissue	VSpdC	60.7	N(60.7, 9.4)
Rapidly perfused tissue	VRpdC	3.71	N(3.7, 0.26)
Blood	VBldC	5.0	U(2.5,10)
Lymph	VLymphC	0.36	U(0.18, 0.72)
			
**Cardiac output (L h** **^-1^ kg^-1^ BW)**	QCC	14	N(13.8, 2.5)
			
**% Cardiac output**			
Liver	QHepartC	6.0	N(6.89, 0.52)
Fat	QFaC	5.0	N(5.3, 0.3)
Gut	QGuC	14.9	U(13.2,16.6)
Stomach	QStC	1.1	N(1.1, 0.08)
Slowly perfused tissue	QSpdC	27.0	N(28.7, 1.91)
Rapidly perfused tissue	QRpdC	42.0	N(43.1, 2.78)
Lymph	QLymphC	0.04	U(0.02,0.08)
			
**Metabolic Clearance (minutes)**			
*In vitro half-life DINCH*	T_½dinch_	30^1^	U(15, 60)
*In vitro half-life MINCH*	T_½minch_	30.53	N(30.54, 2.39)
*In vivo DINCH gut half-life*	T_½dinch_gut_	30	U(15, 60)
**Microsomal protein yield (mg g** **^-1^** **)**			
Hepatic	MPY	34^2^	See **Table 4**
Gut	MPY_gut_	3.9^3^	U(1.95, 7.8)
**Fraction Bound in plasma (proportion)**	Abbreviation	Default Value	Distribution
DINCH	FBDINCH	0.0001249	U(10^-5^, 0.01)
MINCH	FBMINCH	0.0146477	U(0.001, 0.01)
**Gastric emptying (h** **^-1^** **)** **^4^**			
Maximum	k_(max)_	10.2	U(5.1, 20.4)
Minimum	k_(min)_	0.005	U(0.0025, 0.01)
**Absorption (h** **^-1^** **)** **^9^**			
Gut	k_Ga_	25.1	U(12.55, 50.2)
Time taken to consume dose (h)	DRINKTIME	0.25	U(0.125, 0.5)
Fraction of dose taken up into liver	FracDOSE	0.7	See **Table 4**
Fraction of dose taken up into lymphatic system	1- FracDOSE		
Fraction of MINCH metabolised	FracMetab		See **Table 4**
**Urinary production (L h** **^-1^** **)**	Rurine^5^	0.1	See **Table 4**
Creatinine concentration (g L^-1^)	Creat^10^	0.5	See **Table 4**
Urinary excretion rate (h^-1^)	K1	0.15	See **Table 4**

^1^Estimated.

^2^(Barter et al., 2007; Howgate et al., 2006).

^3^(Pacifici et al., 1988; Soars et al., 2002).

^4^(Loizou and Spendiff, 2004).

^5^Volunteer specific (**Table 2**).

The model was parameterized using standard organ and tissue masses and regional blood flow rates (Brown et al., 1997; ICRP, 2002). The mass of the lymphatic system was obtained from Offman et al. (2016) and the lymph flow rate from Thibodeau and Patton (1999).

The tissue: blood PCs and the fractions unbound in plasma were predicted using Log P_ow_ as described in *Prediction of tissue:blood partition coefficients*.

The biotransformation of DINCH to MINCH was described by intrinsic clearance terms in the gut and liver. The biotransformation of MINCH to cx- and OH-MINCH was described by an intrinsic clearance term determined *in vitro* and scaled to *in vivo* described in *Calculation of in vivo clearance*. Likewise, the proportion of MINCH metabolized to cx- and OH-MINCH was estimated as described in *Calculation of fraction metabolized*. The *in vivo* intrinsic clearance of MINCH in the gut was calculated by using the measured *in vitro* hepatic clearance rate and scaled to *in vivo* by replacing hepatic microsomal protein yield with gut microsomal protein yield (Pacifici et al., 1988; Soars et al., 2002) (**Table 3**).

### Statistical Analysis

#### Parameter Distributions

Parameter ranges used for uncertainty analysis and global sensitivity analysis are listed in **Table 3**, where “U,” “N,” and “LN” represent uniform, normal and log-normal distributions respectively. Anatomical and physiological parameter distributions were obtained from the freely available web-based application PopGen (McNally et al., 2014), which is a virtual (healthy) human population generator^3^. A human population of 10,000 individuals, comprising 100% male, white Caucasians, age range 16–65, height range 140–200 cm, body mass indices 18.5–30 was generated to encompass the characteristics of the volunteers that took part in the study of Koch et al. (2013b). Parameter ranges for organ masses and blood flows were set at the 5^th^ and 95^th^ percentiles of the distributions from the virtual population.

The rate of urine (liter per hour) and creatinine (gram per liter) production (**Table 2**) were based upon bounding limits from those observed in the human volunteer study. Both parameters were calculated for each time interval between samples and the mean and standard deviations were derived over those rates. This is more representative of urine production volume over the experimental period whereas a simple average could lead to a single large volume of urine over a single sampling period dominating the rate calculation. This method also permitted the specification of a prior distribution which is not possible with a simple average over the experimental period.

Uniform distributions in **Tables 3** and **4** were assigned to a number of parameters for which there were no available distributions. The minima for the distribution ranges were calculated by dividing the starting or “point” value of the parameter by two and for the maxima multiplying by two.

**Table 4:** Prior distributions and summary statistics from marginal posterior distributions for calibrated parameters for volunteers A and B.

Parameter	Prior	Source	Posterior Distributions	
			Median (90% CI) (Varying Oral Dose)	Median (90% CI) (Fixed Oral Dose)
PORALDOSE	U(0.1,0.9)	Weakly informative	0.30 (0.21,0.41)	0.555
FRACDOSE	U(0,1)	Weakly informative	0.91 (0.68, 0.99)	0.74 (0.53, 0.96)
MPY	N(34,15)	PopGen	39.10 (27.53, 54.16)	38.4 (26.40, 53.12)
K1_OH	U(0.05, 2)	Weakly informative	0.15 (0.13, 0.17)	0.15 (0.13, 0.18)
K1_cx	U(0.05, 2)	Weakly informative	0.10 (0.08, 0.11)	0.10 (0.09, 0.12)
FracMetabMOH	N(0.25, 0.05)	Study data	0.23 (0.19, 0.28)	0.20 (0.16, 0.27)
FracMetabcx	N(0.0475, 0.01)	Study data	0.05 (0.04, 0.06)	0.05 (0.03, 0.06)
Rurine_A,	N(0.104, 0.053)	Study data	0.10 (0.06, 0.16)	0.12 (0.07, 0.18)
Creat_A	N(1.278, 0.605)	Study data	1.27 (0.69, 2.03)	1.40 (0.87, 2.14)
Rurine_B,	N(0.158, 0.086)	Study data	0.12 (0.07, 0.19)	0.14 (0.09, 0.19)
Creat_B	N(0.962, 0.672)	Study data	1.11 (0.70, 2.01)	1.32 (0.87, 2.04)
σ	Improper (∼ σ^-1^)		0.28 (0.25, 0.33)	0.28 (0.25, 0.33)

Point values for parameters for which there was no prior knowledge such as, FracMetab, FracDose, and K1 were determined by choosing a value which provided a reasonable fit to urinary metabolite excretion data.

The 5^th^ and the 95^th^ percentiles of the half-life values used for the calculation of the *in vitro* intrinsic clearance of MINCH were scaled to *in vivo* and used as the minimum and maximum range for *in vivo* clearance.

#### Uncertainty Analysis

A 200 point maxi-min Latin Hypercube Design was created based upon the parameter limits provided in **Table 3**. The PBPK model was run for each of these design points and the concentration response profiles for three outputs studied; DINCH in venous blood and MINCH in venous blood and OH-MINCH in urine. The purpose of this preliminary work was to efficiently assess (based upon a relatively small number of model runs) whether the behavior of the model over the parameter space corresponding to defined parameter limits (**Table 3**) was broadly reasonable. The use of three output measures allowed an evaluation of absorption, uptake and metabolism of DINCH to MINCH, the second step of metabolism of MINCH to cx- and OH-MINCH and elimination of the latter two metabolites in urine. The study of OH-MINCH in urine also allowed an assessment of whether a subset of model runs was consistent with experimental data.

#### Sensitivity Analysis

A two-phased GSA comprising elementary effects screening (Morris test) followed by a variance-based analysis on the retained subset of sensitive parameters was conducted, as described in (McNally et al., 2011; Loizou et al., 2015). In order to perform the Morris screening test and the extended Fourier amplitude sensitivity test (eFAST) GSA the model code was further modified to ensure that logical constraints on mass balance and blood flow to the tissues were met by adopting the re-parameterizations described in Gelman et al. (1996).

In order to simulate the biological monitoring data described in Koch et al. (2013b) the concentration of cx- and OH-MINCH in the urine, Curine was selected as the primary model output, however the venous concentrations of DINCH and MINCH were also studied to assess whether the absorption, uptake and metabolism to MINCH, and the second step of metabolism to cx- and OH-MINCH showed expected sensitivities. For all three outputs the Morris test was performed as described in (McNally et al., 2011). A total of 54 parameters listed in **Tables 1**–**3** were screened and ranked in order of importance (McNally et al., 2011). The top 11 most important parameters were retained in the analysis using eFAST (McNally et al., 2011). The variance-based eFAST analysis was conducted over a time period of 50 h to cover the entire biological monitoring sampling period described in (Koch et al., 2013b).

#### Calibration

**Figure 4:**
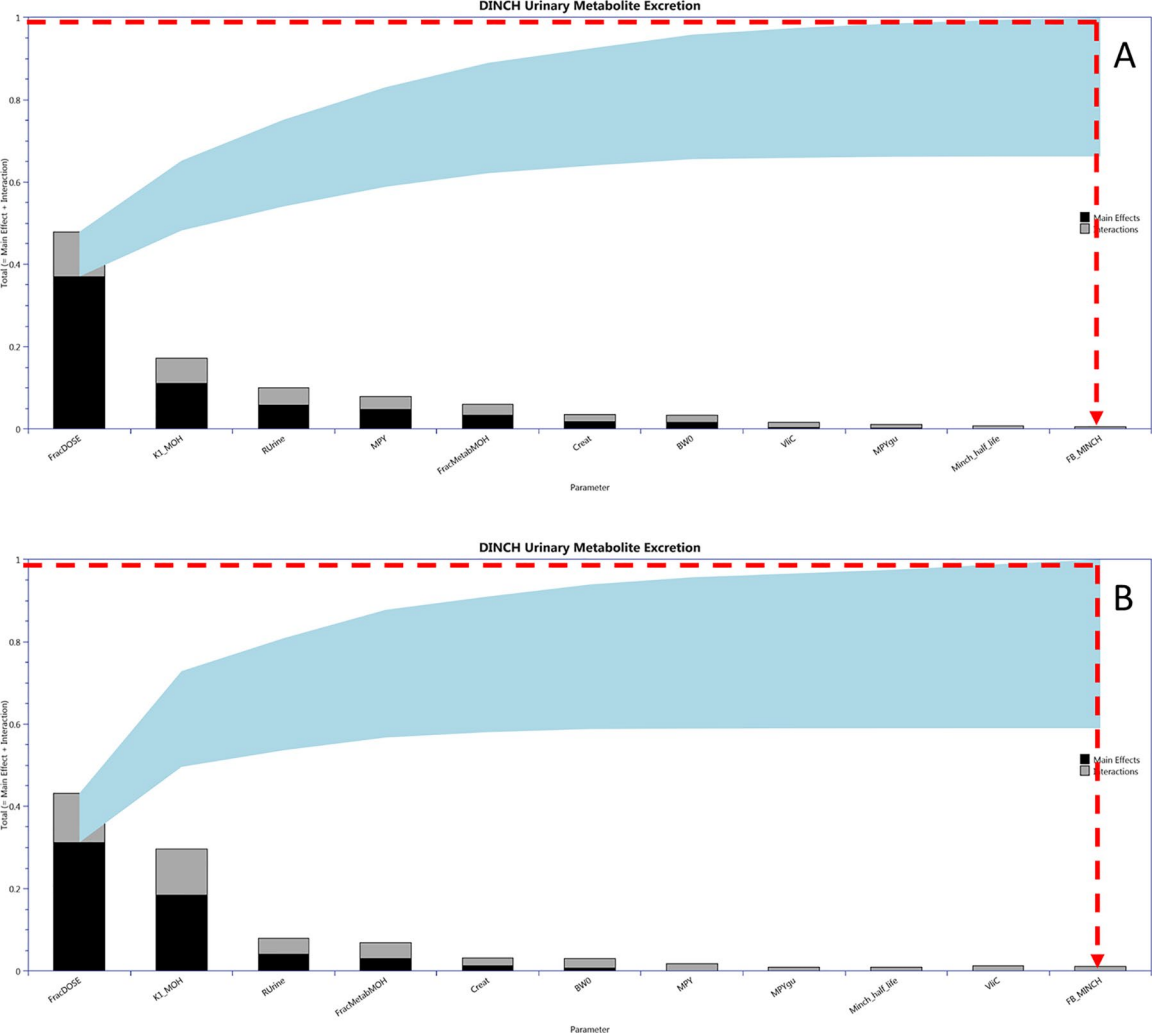
Lowry plots of the 11 most influential parameters governing C_urine_ variance at **(A)** 2 h and **(B)** 10 h. FracMetab, FracDose, Creat, RUrine, BW, MPY, and K1 were the most important parameters throughout the entire 50 h simulation and accounted for almost 100% of variance in C_urine_. In order to account for 100% variance the red broken line was drawn from 1 (100% variance point) on the y axis to the ribbon and then vertically down to the x axis. All parameters to the left of the vertical line account for all the variance in C_urine_.

Calibration of a subset of uncertain parameters in the PBPK model using a dataset of metabolites measured in timed urine samples following ingestion of DINCH (Koch et al., 2013b) was attempted. The subset of sensitive parameters taken forward to calibration was based upon results of variance-based GSA of urinary concentrations of metabolites. This was conducted by generating a Lowry plot (McNally et al., 2011) and drawing a line from the 100% of the total effects point on the y-axis to the point of contact with the ribbon where a perpendicular line was drawn down to the x-axis (**Figure 4**). All the parameters to the left of the perpendicular were selected for parameter estimation. Since BW was a known measured parameter it was not included in the parameter estimation. Instead it was fixed at the measured value along with the remaining 49 relatively insensitive parameters. PORALDOSE represented the dose of DINCH administered orally and was included in the set of parameters to be estimated in the initial calibration—this allowed us to study the precision with which the known ingested dose could be estimated, based upon urinary metabolite data, a process referred to as reverse dosimetry. The ingested dose was subsequently fixed, with a further calibration of the unknown sensitive parameters attempted.

The Bayesian approach described in McNally et al. (2012) was followed. This requires the specification of a joint prior distribution for sensitive parameters, which is refined through a comparison of model predictions (corresponding to a given parameter set) and measurements within a statistical model. The resulting parameter space that is consistent with the prior specification and measurement data is the posterior distribution.

Marginal prior distributions for model parameters are described in **Tables 4** and **5**. For most of the parameters, weakly informative prior distributions were specified, which provided conservative upper and lower bounds for parameters, however within these ranges the prior distributions were flat. A mean and standard deviation for the rate of urine production and creatinine concentration based upon the biological monitoring from the study of Koch et al. (2013b) were initially estimated for each study participant. Furthermore, a correlation of -0.7 between Rurine and Creat was estimated from the data of Koch et al. (2013b). Based upon means, standard deviations and this correlation a multivariate normal prior distribution for these two parameters was specified for each study of the study participants. An improper prior distribution was assumed for σ.

**Table 5:** Prior distributions and summary statistics from marginal posterior distributions for calibrated parameters for volunteer C.

Parameter	Prior	Source	Median (90% CI) (Fixed Oral Dose)
PORALDOSE	U(0.1,0.9)	Weakly informative	0.606
FRACDOSE	U(0,1)	Weakly informative	0.39 (0.19, 0.92)
MPY	N(34,15)	PopGen	18.72 (17.09, 27.31)
K1_OH	U(0.05, 2)	Weakly informative	0.66 (0.45, 0.86)
K1_cx	U(0.05, 2)	Weakly informative	0.37 (0.27, 0.56)
FracMetabMOH	N(0.202, 0.05)	Study data	0.23 (0.17, 0.28)
FracMetabcx	N(0.049, 0.01)	Study data	0.05 (0.04, 0.07)
Rurine_OH	N(0.190, 0.242)	Study data	0.10 (0.06, 0.19)
Creat_OH	N(1.278, 0.831)	Study data	1.17 (0.47, 1.99)
σ	Improper (∼ σ^-1^)		0.50 (0.42, 0.64)

**Figure 5:**
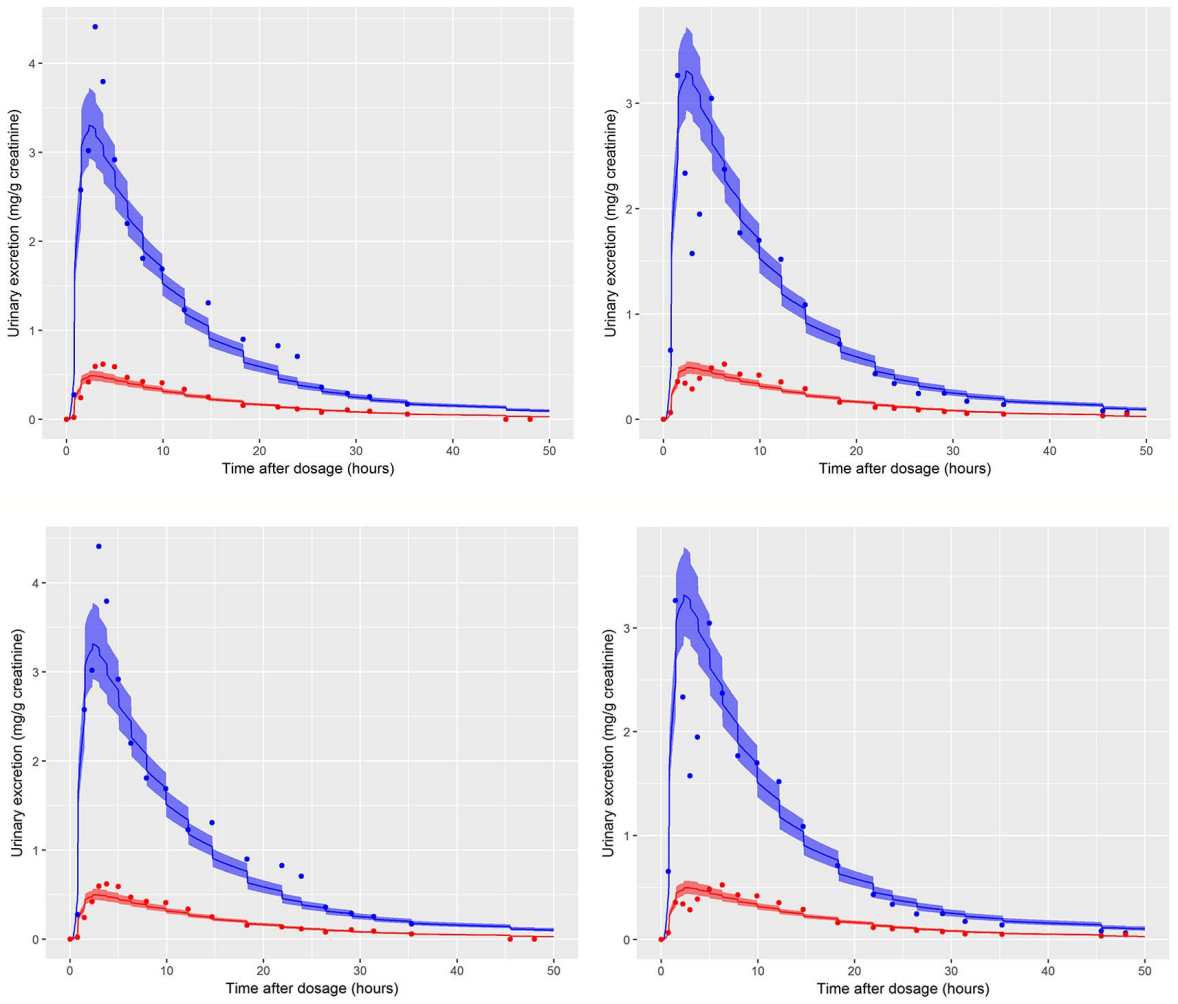
Simultaneous fit to both cx- (red line and symbols) and OH-MINCH (blue line and symbols) using a common set of calibrated parameters showing the 90% credible interval for volunteers A and B The upper panels show the fit when estimating PORALDOSE and lower panels when PORALDOSE is fixed.

Preliminary investigations attempted calibrations based upon measurement data on a single individual and single metabolite (either OH-MINCH or cx-MINCH). The final calibration model (6) used data from two individuals^4^ (A and B) and both metabolites—the fractions metabolized to OH-MINCH or cx-MINCH and separate elimination rates, K1-OH and K1-cx, respectively were therefore included as parameters (**Table 4**, **Figure 5**) . A log-normal calibration model (7) was specified for this comparison, where *y* *_ijk_* and *µ* *_ijk_* denotes the measurements and PBPK model predictions respectively of the concentration of metabolite *i* (OH-MINCH and cx-MINCH) in individual *j* (individuals A and B) at times *k* = 1..*T*. The uncertain parameters in the PBPK model are denoted *θ*, and *σ* is a statistical parameter quantifying the disagreement between predictions and measurements

$$f(y|\theta ,\sigma )=\prod_{i=1}^{T}\frac{1}{2\pi \sigma^{2}}\exp\left[-0.5{\left(\frac{\log(y_{i})-\log(\mu_{i})}{\sigma }\right)}^{2}\right]$$

Inference for model parameters *θ* and statistical parameter *σ* was made using a Markov Chain Monte Carlo (MCMC) algorithm (bespoke Metropolis–Hastings algorithm coded in R). The MCMC algorithm was burnt in for 1,000 iterations and sampling was conducted for a further 10,000 iterations for each model. Every 10^th^ sample was retained for analysis. Checks were made to ensure convergence of the chains and that the auto-correlation was reasonable.

### Software

The PBPK model was written in the R language (R Development Core Team, 2008) and run using RStudio (RStudio Team, 2016).

PBPK models were solved using the deSolve package of R. GSA of model outputs (Morris screening test and eFAST) were conducted using the Sensitivity package of R. Reshaping of data and plotting was performed using the reshape and ggplot2 packages, respectively. The md2c package was used to induce rank correlations in samples (Wickham, 2007; Pouillot and Delignette-Muller, 2010; Soetaert et al., 2010; Pujol and Iooss, 2015). The main effects and total effects (McNally et al., 2011) were computed at each time point and parameter sensitivities were studied over this period using Lowry plots generated as described in McNally et al. (2011).

All plots were created using R and gglot2 (R Development Core Team, 2008; Wickham, 2016).

#### Hardware

The computer used in this study was a Dell Optiplex 9020 with an Intel(R) Core ™ i5-4590 CPU @ 3.30 GHz with 8.00 GB RAM running Windows 7 Enterprise Service Pack 1.

## Results

### *In Vitro* Clearance

A whole liver intrinsic clearance value of 184.3 l h^-1^ was calculated using Equations 3 and 4 with the half-life of 30.53 min for the *in vitro* clearance of MINCH measured in pooled human hepatic microsomes (**Figure 2** and **Table 2**) This value is less than two-fold greater than a whole liver intrinsic clearance value of 101.9 l h^-1^ for MEHP an analogue monoester metabolite of DEHP which is initially metabolized to 5-OH MEHP and 5-carboxy MEPP (calculated from **Table 4** in Choi et al. 2013).

### *In Silico* Predicted Parameters

A Log P_ow_ of 9.77 was predicted for DINCH. This indicates a four orders of magnitude higher lipophilicity than MINCH which has a predicted Log P_ow_ of 5.17 (**Table 1**). The very high lipophilicity of DINCH means that insufficient concentrations were present in the predominantly aqueous reaction medium for the *in vitro* measurement of clearance. Hence, the *in vitro* clearance of MINCH only was possible.

The tissue:blood PCs, in turn were predicted from the predicted Log P_ow_ for DINCH and MINCH where the adipose:blood PC for DINCH was considerably higher than for MINCH. This is consistent with adipocytes being composed primarily of cells specialized in storing energy as fat. However, the other tissue:blood PCs for MINCH were higher than for DINCH reflecting the heterogeneity of lipid, protein and water contents of the various of cell types within a given organ or tissue.

The plasma fractions unbound for both DINCH and MINCH were also calculated from the predicted Log P_ow_. The unbound fraction in plasma for MINCH was 145 fold higher than for DINCH (**Table 1**).

### Uncertainty and Sensitivity Analysis

Prior to the analysis of HBM data a thorough evaluation of model structure, in terms of the code and the mathematical equations used to describe the chemical and biological mechanisms proposed as relevant to DINCH and MINCH biokinetics was conducted. The aim of this exercise was to confirm that the general behavior of the model was consistent and biologically plausible. The initial analysis focussed on the concentration-time profiles of DINCH and MINCH in plasma in addition to the main model output, Curine, the excretion of urinary metabolites. The results from this exercise are available in the **Supplementary Materials**. In addition, estimates of tissue dosimetry in plasma, liver and adipose, which may inform the selection of concentrations for *in vitro* studies, are also presented in the supplementary materials.

The elementary effects (Morris) screening suggested that the concentration of OH-MINCH (and cx-MINCH) expressed relative to creatinine in urine was primarily sensitive to parameters influencing the urinary elimination rate of metabolite (K1), the half-life (T_½minch_) and therefore intrinsic clearance, the fractions of MINCH metabolized to OH-MINCH and cx-MINCH (FracMetabOH), the dilution of metabolite in urine (Rurine, Creat) and the fraction of DINCH entering the lymphatic system (FracDose) following ingestion (DRINKTIME). The computationally cheaper elementary effects screening cannot distinguish between interactions and non-linearities therefore, the top 11 parameters identified from the screening were taken forward to the second stage of GSA and studied using the eFAST variance based analysis.

**Figure 4** is a Lowry plot showing the total effect of a parameter *S* *_T_*, which is comprised the main effect *S* *_M_* (black bar) and any interactions with other parameters *Si* (grey bar) given as a proportion of variance (McNally et al., 2011). The ribbon (light blue), representing variance due to parameter interactions, is bounded by the cumulative sum of the main effects (lower bound) and the minimum of the cumulative sum of the total effects (upper bound). FracMetab, FracDose, Creat, RUrine, BW, MPY, and K1 were the most important parameters throughout the entire 50 h simulation and accounted for almost 100% of variance in C_urine_. In order to reduce computational overhead, Minch_T_½_ was excluded from the parameter estimation exercise because it decreased further with time from a point of very low significance. Therefore, the parameter sensitivities at 2 and 10 h post exposure shown in **Figure 4** are representative example plots. The results from the more computationally demanding eFAST technique indicated that interactions between parameters as opposed to non-linearities drove the underlying variability in the model output (the greater the width of the ribbon or the greater height of the light grey bars of the Lowry plot indicate greater effects of interactions between parameters).

### Calibration

Summary results from calibration using urinary OH-MINCH and cx-MINCH concentrations from two volunteers (A and B) are tabulated in **Table 4**; marginal posterior medians and 90% credible intervals for each parameter are provided for the cases where the external dose [PORALDOSE (milligrams per kilogram)] was treated as unknown and included in the subset of parameters to be calibrated, and where PORALDOSE was fixed. A numerically derived median and 90% credible interval^5^ for the concentration-response profile is shown for individuals A and B in **Figure 5** under each of the calibrations described above.

The satisfactory fit to both urinary cx- and OH-MINCH concentrations from two volunteers (A & B) simultaneously, using a common set of calibrated parameters (except for Rurine and Creat, which were individualized model parameters) supports that the structure of the model, albeit a simplified description of reality, was reasonable. Also, the anatomical, physiological, biochemical, and physicochemical parameter values used, including those predicted using algorithms and mathematical relationships such as PCs, plasma fraction unbound and fraction metabolized provided a satisfactory simulation of the pharmacokinetics of DINCH and MINCH (**Figure 5**).

Marginal posterior distributions for MPY, and the individualized Rurine and Creat parameters and the two elimination rates were broadly consistent over the two calibrations (**Table 4**).The calibrated model suggests a more rapid elimination of cx-MINCH in urine compared to OH-MINCH.

For the initial calibration exercise, PORALDOSE was not fixed to the known administered dose of 0.555 mg/kg BW/day which was in excess of the upper limit of the 90% credible interval^6^ (0.21, 0.41) (**Table 4**). However, in the calibration exercise where PORALDOSE was fixed at 0.555 mg/kg BW/day almost identical results were obtained, in terms of the numerically derived central estimates and 90% credible interval for the concentration- response (**Figure 5** volunteer A estimated dose and volunteer A fixed dose respectively, and Figure volunteer B estimated dose and volunteer B fixed dose respectively). This apparently surprising finding can be attributed to the influence of prior distributions for secondary MINCH metabolites OH-MINCH and cx-MINCH. These priors were estimated from the metabolite data for the three volunteers, and although intended to be relatively weak, were sufficient to discriminate between parameter sets which provided a similar quality of fit to the HBM data. Specifically, the smaller proportions of MINCH metabolized to OH-MINCH and cx-MINCH in the calibration with fixed PORALDOSE were less consistent with the prior distribution (**Table 4**). Uniform distributions for OH-MINCH and cx-MINCH over similar ranges to the normal distributions used in calibration would have resulted in a wider marginal posterior for PORALDOSE consistent with the administered dose of 0.555 mg/kg BW/day. This result suggests that the sensitivity of results to prior distributions should be considered when information on the associated model parameters is weak. However, a consequence of fixing PORALDOSE was a notable reduction in the parameter FracDOSE, reflecting a greater amount entering the lymphatic system. This suggested that simulations that corresponded to a negligible amount of DINCH entering the lymphatic system were no longer consistent with the HBM data.

**Figure 6:**
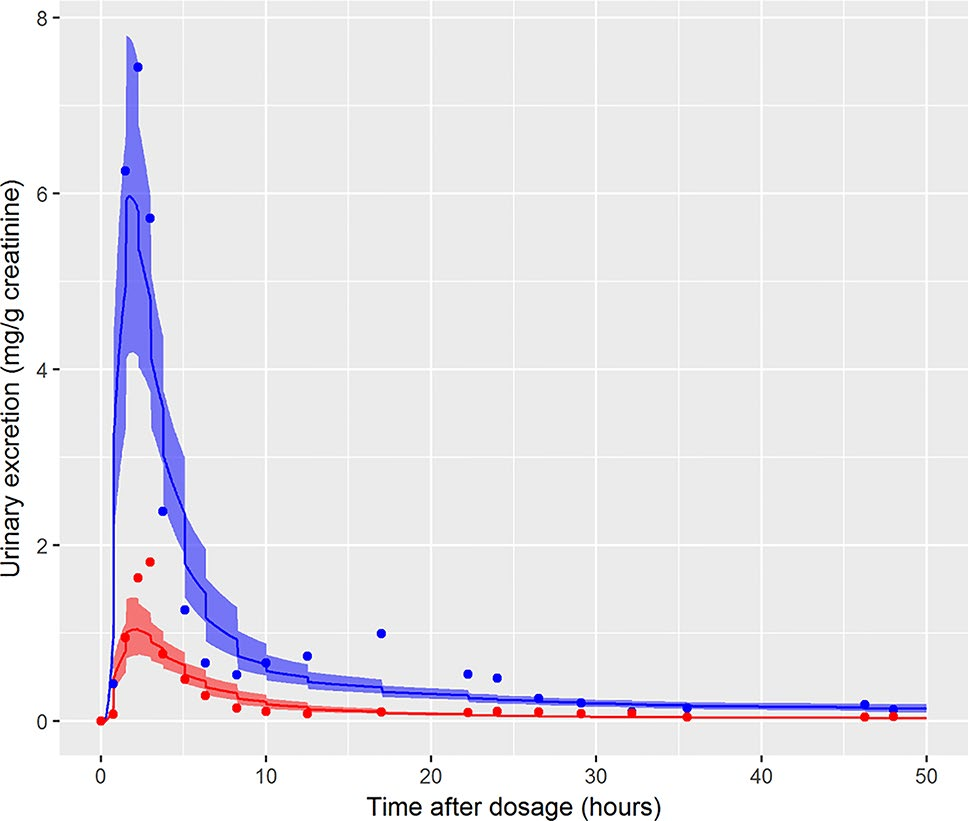
Simultaneous fit to both cx-(red line and symbols) and OH-MINCH (blue line and symbols) using a common set of calibrated parameters showing the 90% credible interval for volunteer C with fixed PORALDOSE.

The data from volunteer C were excluded from the simultaneous model calibration because the urine volumes were unusually large and the creatinine concentrations unusually low at 45, 90, 135, and 180 min post oral administration (volunteer C drank a large volume of water at the start of the study—Dr. Holger Koch personal communication). This resulted in the first three measurements of both cx-MINCH and OH-MINCH (milligrams per gram creatinine) being comparatively high and the fourth comparatively low concentrations (**Figure 6**). As a consequence the constant rates of urine and creatinine production assumed in the PBPK model were clearly inconsistent with measurements from this volunteer. However, calibration using the volunteer C HBM data was attempted in order to investigate the consequences of calibrating to unusual data which were inconsistent with an assumption (constant rates of urine and creatinine production) in the underlying PBPK model. Results from the fixed PORALDOSE calibration are reported in **Table 5**. The median and 90% credible interval for the concentration–response (calculated as described previously) are shown in **Figure 6**. Surprisingly, the broad trend of the data was reasonably well fitted, although with greater residual error (**Figure 6**, see sigma values in **Table 5**). However, in order to fit the HBM data the fraction of dose taken into the lymphatic system was notably higher, MPY was notably lower and the elimination rates of OH-MINCH and cx-MINCH in urine were notably slower (corresponding to larger K1 values) compared with calibration to data from volunteers A and B (**Tables 4** and **5**).

## Discussion

In this work we described the development of a PBPK model for the oral ingestion of the plasticizer DINCH and its metabolite MINCH and the subsequent biotransformation of MINCH to cx-MINCH and OH-MINCH. Model structure was informed by properties of the chemical and experimental work, with central estimates of model parameters provided by *in vitro* hepatic intrinsic clearance scaled to *in vivo* whole liver intrinsic clearance, *in silico* predicted PCs and plasma unbound fractions of DINCH and MINCH, and population databases. The performance of the model was investigated using uncertainty and sensitivity analysis techniques and a small number of sensitive parameters were calibrated using data from urine voids provided by a human *in vivo* biomonitoring study. GSA confirmed that the accuracy of these *in vitro* and *in silico* derived parameter values were not important in determining variance in urinary metabolite concentrations. Notably for this model the sensitive parameters were primarily structural (such as elimination rates and fractions metabolized via different pathways), thus representing reducible sources of uncertainty. Apart from differences in the rate of uptake from the gut, substantial inter-individual differences in the subsequent distribution and elimination of DINCH are inconsistent with the model. Following calibration the principle uncertainties were in the fractions of dose entering the lymphatic system (FracDose) via the gut and in the fractions of MINCH that were bio-transformed (FracMetabOH, FracMetabcx) and subsequently eliminated as cx-MINCH and OH-MINCH in urine. Alternative sources of information on these parameters, quantified through informative prior distributions, could in principle, further reduce the uncertainties in the calibrated model.

In the remainder of the discussion we comment on the process of calibration, the subsequent verification of model performance and comment on the implications for calibration problems requiring reverse dosimetry when dose is genuinely unknown, and when a surrogate for the *in vivo* human (human on a chip, *in vitro* systems etc.) is utilized; this has particular relevance for the paradigm where it is envisaged that animal data will not be utilized in RA and/or pharmacokinetic models are used to enhance toxicological assessment based on “read across” (Ellison, 2018).

In the results section the influence of the informative prior distributions for cx-MINCH and OH-MINCH was briefly discussed—the priors used favoured simulations with PORALDOSE below the known administered dose. In preliminary calibration models (results from this phase of work are not reported) non-informative uniform (0, 1) priors were specified for the fraction of MINCH bio-transformed and subsequently eliminated in urine as cx-MINCH and OH-MINCH. However, due to large correlations between FracMetabOH and FracMetabcx and the assumed unknown PORALDOSE, the marginal posteriors for all three parameters were unrealistically wide (lower dose and higher fractions metabolized to OH-MINCH and cx-MINCH and high dose with small fractions metabolized to OH-MINCH and cx-MINCH were consistent with concentration- response relationships in these two metabolites). It was therefore important to constrain FracMetabOH and FracMetabcx in the final calibration models—the priors (as given in **Tables 4** and **5**) were therefore derived from the study data of Koch et al. (2013b), as described in *Calculation of fraction metabolized* (**Table 2**). An alternative strategy would be to extend the PBPK model in order to describe all downstream metabolites of DINCH.—Whilst in principle the PBPK model could be extended and calibrated using a wider pool of concentration- response data from the study of Koch et al. (2013b), not all downstream metabolites are specific to DINCH for example cyclohexane-1,2-dicarboxylic acid. Therefore, biologically realistic constraints on the parameters FracMetabOH and FracMetabcx based upon the full spectrum of metabolite data held on each individual represents a reasonable and more practical approach. The importance of prior distributions which effectively constrain model parameters has been previously recognized (McNally and Loizou et al., 2015), although this was in the context of tighter bounds on human variability rather than structural parameters.

**Figure 7:**
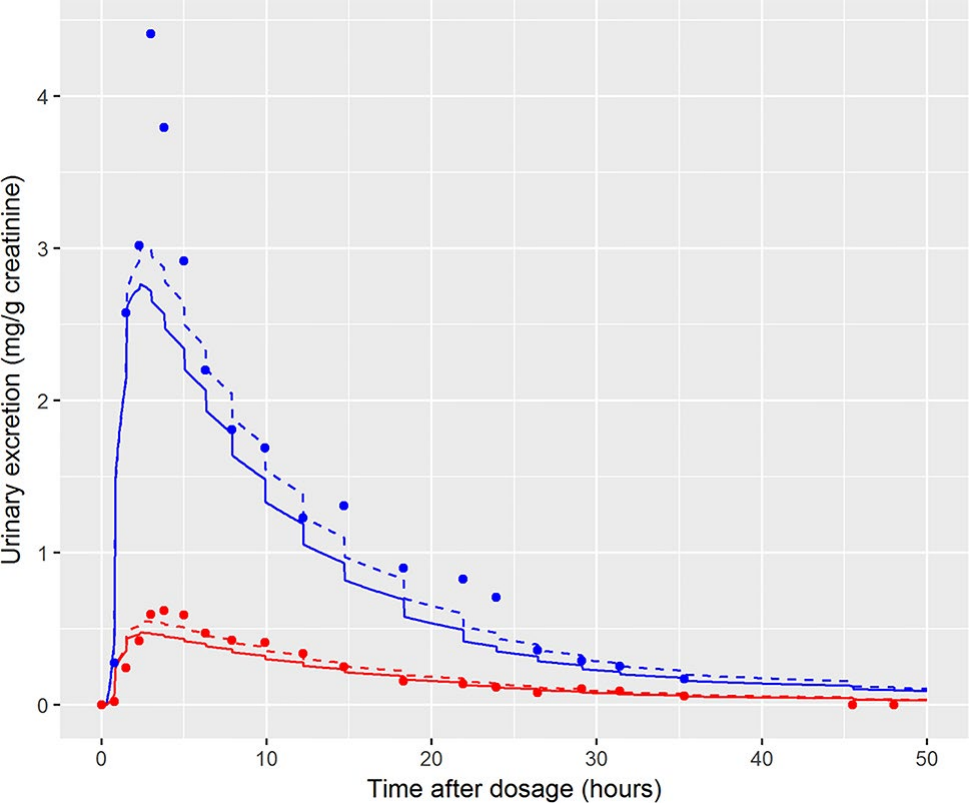
Comparison of the fit of two draws from the joint posterior distribution to demonstrate the wide range of PORALDOSE consistent with data of volunteer A [0.22 mg kg^-1^ (solid line) and 0.4 mg kg^-1^ (broken line)], [cx-(red lines and symbols) and OH-MINCH (blue lines and symbols)].

In the initial calibration model the administered dose (PORALDOSE) was treated as unknown and therefore included in the group of calibration parameters, in order to investigate how well this dose could be estimated from HBM data, after accounting for other parameter uncertainties. Results from this calibration exercise showed that a relatively wide range (wide for model calibration using HBM data) of PORALDOSE (90% credible interval of 0.21 to 0.41 mg/kg) was consistent with urine voids: the fit of two such parameter sets (selected from MCMC output) to bio-monitoring data from individual A corresponding to PORALDOSE of 0.22 and 0.40 mg/kg (approximately 20 and 40 mg of ingested DINCH) are shown in **Figure 7** and indicate similar concentration-response profiles despite the difference in external dose. A pertinent question based upon this finding is “what are the key differences in the distribution of DINCH and its metabolites within the body, which correspond to these different administered doses?” The principle parameter difference (other than dose) corresponding to the two simulations shown in **Figure 7** was in the fraction of dose entering the lymphatic system and hence by-passing first-pass metabolism in the liver before entering and mixing into venous blood. For the lower value of dose a very low fraction, 4%, entered the lymphatic system, whereas at the higher dose 30% went down this route (correlations in the MCMC output suggest these two runs are representative of the model behavior). An examination of model output from these two runs revealed that the additional 20 mg of dose was accounted for by DINCH in the lymphatic system, which slowly decreased through the simulation, and as DINCH bound to protein within the plasma. The critical mechanism that facilitates the uptake of larger doses, and which therefore is more consistent with the known ingested dose, is the by-passing of first-pass metabolism and subsequent binding of parent chemical to protein within plasma. Due to the very high bound fraction for the parent chemical, larger doses of DINCH are consistent with the near zero concentrations of OH-MINCH and cx-MINCH observed in urine voids after 35 h. An important finding from this exercise is that an adequate fit to HBM data could be achieved without modeling the lymphatic system; however the external dose required to achieve such a fit to HBM data was less than half the known administered dose. This has important consequences for reverse dosimetry applications where the external dose is genuinely unknown: an adequate fit to an *in vitro* or HBM dataset is insufficient in itself to validate the structure of a PBPK model. Furthermore, an adequate fit to the data from individual C was achieved (**Figure 6**) despite the significant variability in the urine volumes and creatinine concentrations from this volunteer, as described in results. However, the required parameters to achieve a good fit to HBM data were inconsistent with results from individuals A and B. Through the application of sensitivity analysis, which demonstrated the sensitivity of the model outputs under study to the rate of urine production and creatinine concentrations, and a careful analysis of the HBM data prior to calibration the problem with individual C was identified prior to attempting calibration. Our results demonstrate that study data need to be verified and consistency with the internal assumptions of a PBPK model assessed. The conclusion from these observations are that the quality of HBM can have a significant impact on parameter estimates i.e., reverse dosimetry. The prediction of inaccurate posterior distributions can be minimized by determining and using plausible physiological, biochemical and physicochemical constraints around prior distributions for sensitive parameters.

A simplistic description of the lymphatic system was coded into the model. We view this as a physically informed mechanism for a fraction of the ingested dose to avoid first pass metabolism in the liver, prior to mixing in venous blood. The mechanism for mixing is particularly simplistic—this is an area where the model could be improved if accurate modeling of distribution in the lymphatic system was important. However, due to the very high fraction bound to protein, the rate of stripping from plasma rather than the very slow flow rate of the lymph was the dominant factor in the rate of elimination of downstream metabolites of DINCH.

**Figure 8:**
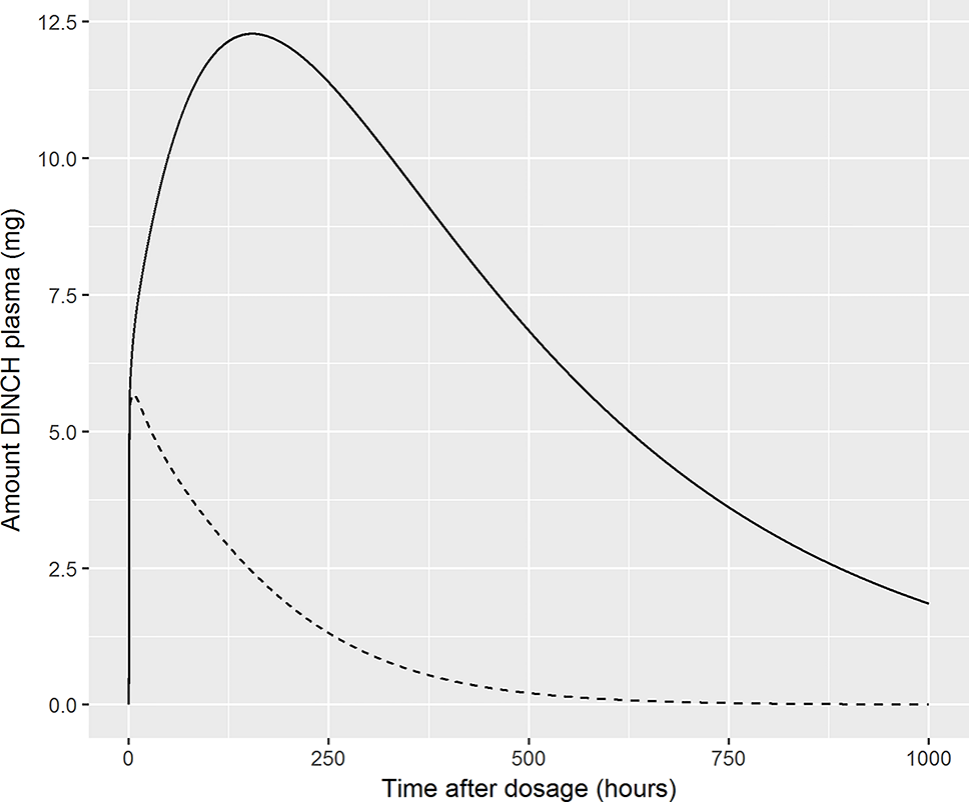
Sensitivity of plasma fraction bound for DINCH. The amount (milligram) of DINCH in plasma over a 1,000 h period is shown for the posterior mode parameter set (volunteer A) and default binding. A similar simulation is shown for a 10 fold reduction (corresponding to decreasing from 99.99 to 99.9% binding) in fraction bound: the small change in absolute value (10-fold multiplicative difference) results in an important change in the duration of clearance from plasma following ingestion [99.99% (solid line), 99.9% (broken line)].

Finally, we comment on the bound fraction of DINCH, which was estimated based upon equation 5. The bound fraction of DINCH was not calibrated in this current work since sensitivity analysis, over the very narrow limits considered for this parameter when model output was Curine, indicated the concentrations of OH-MINCH and cx-MINCH in urine were insensitive to bound fraction of DINCH. However, the amount (and concentration) of DINCH in plasma and the rate of clearance following ingestion was investigated following calibration of the model when model output was venous concentration of DINCH. In **Figure 8** the amount (mg) of DINCH in plasma over a 1,000 h period is shown for the posterior mode parameter set (individual A) and default binding. A similar simulation is shown for a 10 fold reduction (corresponding to decreasing from 99.99 to 99.9% binding) in fraction bound: the small change in absolute value (10-fold multiplicative difference) results in an important change in the duration of clearance from plasma following ingestion. An important conclusion from this work is that the consequences of the model should be studied following calibration, the credibility of the compartmental predictions scrutinized, and opportunities for refining the model through either expert knowledge or experimental data should be considered. This means that evaluation of model performance should involve the sensitivity analysis of a range of model outputs, including compartments for which there are no HBM data, in this case venous blood concentrations of DINCH and MINCH. Expert judgement should then be used to interpret the impact of these analyses on those model outputs for which there are HBM data. For the model for DINCH there is considerable added value in refining the estimates of four parameters, FracDOSE, FBDINCH, FracMetabMOH, and FracMetabcx, governing the fraction entering the lymph, the fraction of DINCH bound to protein and the fractions of MINCH bio-transformed and excreted as OH-MINCH and cx-MINCH.

## Data Availability Statement

The datasets analyzed in this manuscript are not publicly available. Requests to access the datasets should be directed to Professor Holger M Koch, koch@ipa-dguv.de.

## Ethics Statement

The studies involving human participants were reviewed and approved by Ruhr-University Bochum (Reg. No.: 3866-10). The patients/participants provided their written informed consent to participate in this study.

## Author Contributions

GL and KM developed the PBPK model, analyzed and interpreted the data. CS measured *in vitro* clearance in human microsomes and analyzed the data. All three authors made significant contributions to the manuscript.

## Funding

This work was supported by The Members of European Plasticisers (Grant No: T 50_UK HSL-02-471-0000-07-T50), a sector group of CEFIC, the European Chemical Industry Council and VinylPlus^®^, the voluntary sustainable development programme of the European PVC industry.

## Conflict of Interest

The authors declare that the research was conducted in the absence of any commercial or financial relationships that could be construed as a potential conflict of interest.

## Footnotes

1
http://www.acdlabs.com (accessed 5th July 2018).

2
http://www.acdlabs.com (accessed 5th July 2018).

3 (http://xnet.hsl.gov.uk/popgen) (accessed 21th March 2019).

4 Urinary data from participant C was judged to be unreliable (**Figure 6**).

5 The 1,000 retained parameter sets from the posterior were run through the PBPK model with results ordered at each time point and percentiles read off.

6 Confidence interval derived using a Bayesian approach.
