# Supplementary material for: Development, Testing, Parameterization, and Calibration of a Human Physiologically Based Pharmacokinetic Model for the Plasticizer, Hexamoll® Diisononyl-Cyclohexane-1, 2-Dicarboxylate Using In Silico, In Vitro, and Human Biomonitoring Data
Source: Front Pharmacol. 2019 Nov 29;10:1394. doi: 10.3389/fphar.2019.01394 (PMC6897292; doi:10.3389/fphar.2019.01394)
Supplement: Supplementary file 1 [file DataSheet_1.pdf]

Supplementary Materials

Morris Screening

Concentration response profiles corresponding to the LHD are shown for the concentration of DINCH and MINCH in venous blood (mg/L) and the concentration of OH-MINCH and cx-MINCH expressed relative to creatinine in urine (mg/g creatinine) in, Figures S1 to S4 respectively. These simulations indicated a maximum concentration of both DINCH and MINCH in venous blood were rapidly achieved following ingestion and fell rapidly following a sharp peak. Apart from the magnitude of the peak, there was little qualitative difference on the concentration response profile over the runs (Figures S1 and S2). Simulations of metabolites in urine (Figure S3 and S4) showed larger differences in the timing and magnitude of peak concentration of metabolite in urine and in the rate of the subsequent decline in concentration. Overall, the simulations indicated the qualitative behaviour over the model was reasonable over the ranges of the model inputs, which serves as a check on the coding of the model and the assumed distributions and ranges for parameters, and indicated that a subset of runs were broadly consistent with profiles of the HBM data.

A subset of results from the elementary effects screening analysis (Morris Test) is given in Tables S1 to S3 and Figures S5 to S7 of supplementary material. Results from the sensitivity analysis of DINCH in venous blood at 0.5 and 2 hours following ingestion are shown in Table S1and Figure S5; results from sensitivity analysis of MINCH in venous blood at 1 and 3 hours following ingestion are shown in Table S2 and Figure S6; and results from sensitivity analysis of OH-MINCH expressed relative to creatinine in urine at 2 and 10 hours following ingestion in Table S3 and Figure S7. The μ^* and σ metrics are qualitative measures of the overall effect of a parameter on model output (μ^*) and on the degree of non-linearity of interactions with other parameters (σ). The points separated away from the origin in Figures S5 to S7 indicate the most influential parameters on the respective model outputs.

The concentration of DINCH in venous blood was particularly sensitive to parameters that influenced the rate of uptake from the gut and rate of metabolism of DINCH and to the fraction entering the

lymphatic system and thus by-passing first-pass metabolism (DRINKTIME, FracDose, Dinchhalf_life). Similarly the concentration of MINCH in venous blood was particularly sensitive to parameters influencing the rate of uptake (DRINKTIME), the rate of metabolism of MINCH (Minchhalf_life) and the fraction entering the lymphatic system (FracDose). These two measures (DINCH and MINCH in venous blood) were not analysed further - the elementary effects screening was sufficient to indicate these model outputs showed the effected sensitivities to model parameters and the model was behaving reasonably (with respect to concentration-response profiles for these model outputs).

MINCH Tissue Dosimetry

In order to help inform in vitro studies the model was used to estimate concentrations of MINCH in plasma, liver and adipose tissue. The model was exercised assuming an 85 kg male and a 70 kg female receiving a single daily intake dose and simulated for 30 hours. The plasma concentration was calculated by subtracting the concentration of MINCH in red blood cells from total (bound + free) plasma concentration. The peak and average concentrations are reported in Tables S3 and S4.

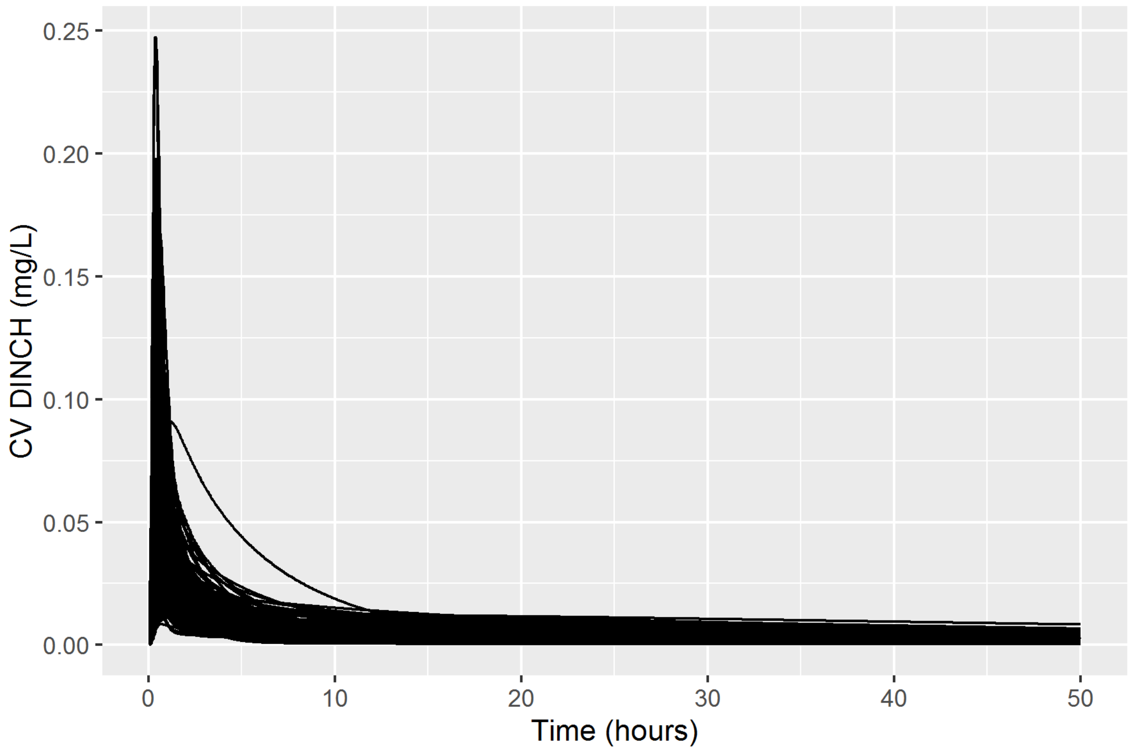

Figure S1: A comparison of 200 concentration-response profiles for the concentration of parent chemical (DINCH) in venous blood

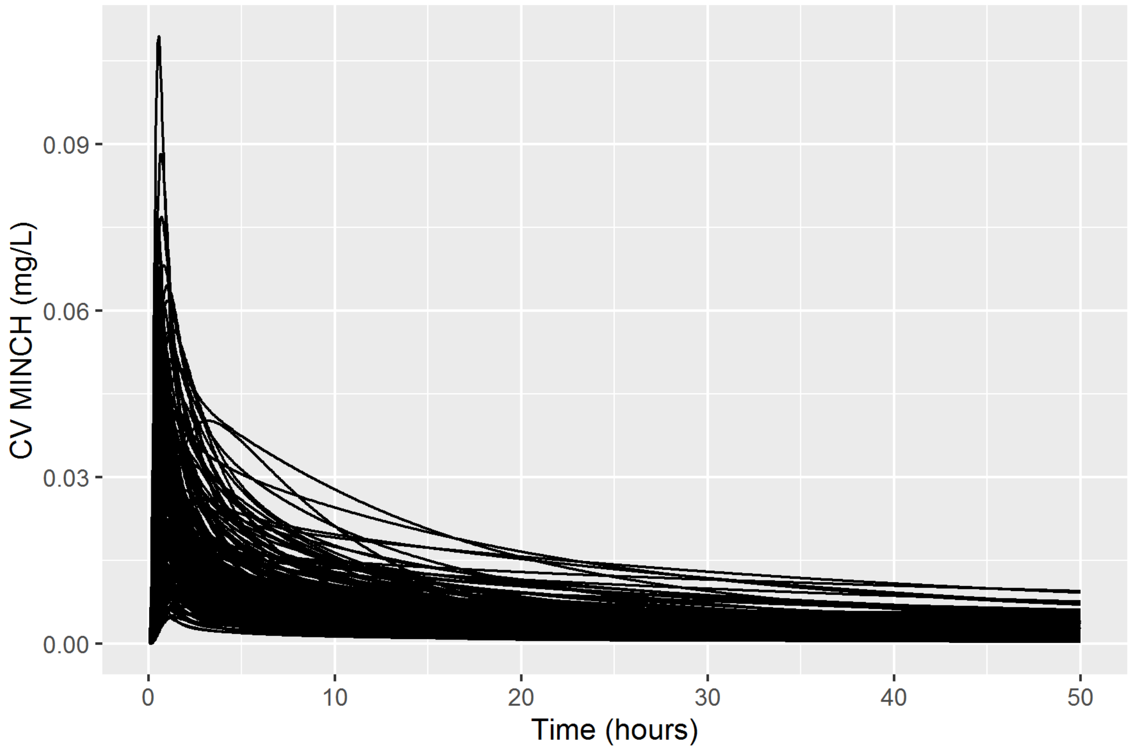

Figure S2: A comparison of 200 concentration-response profiles for the concentration of metabolite MINCH in venous blood

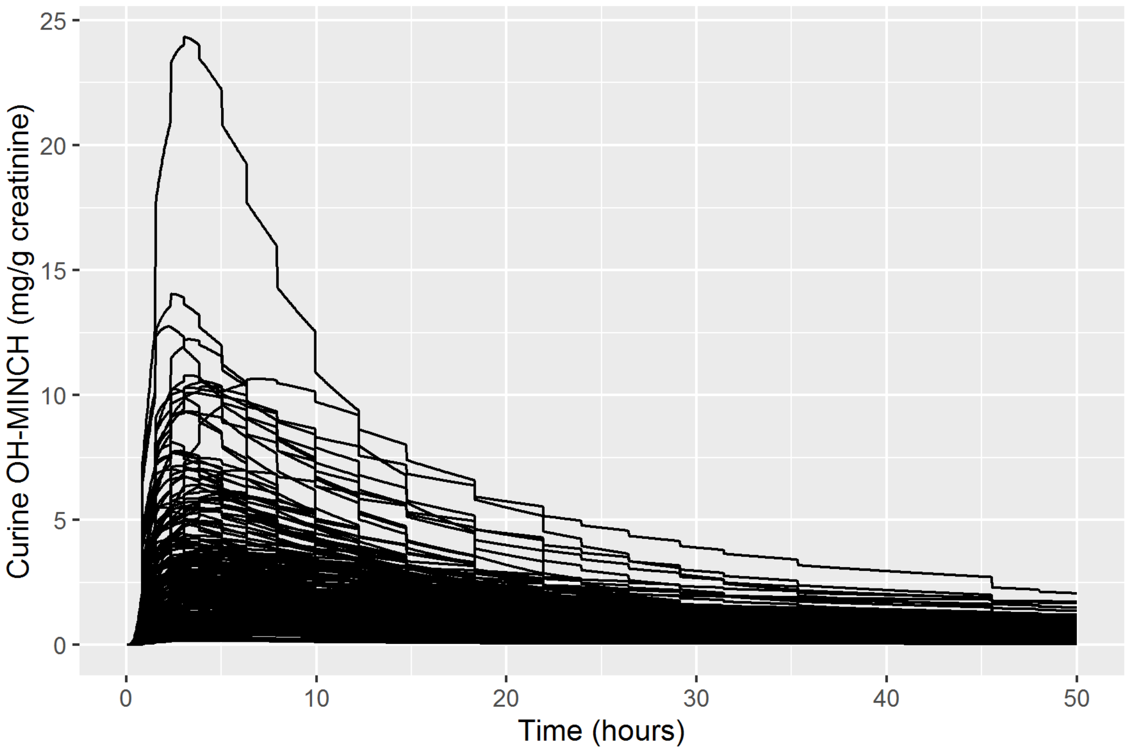

Figure S3: A comparison of 200 concentration-response profiles for the concentration of metabolite OH-MINCH expressed relative to creatinine concentration in urine

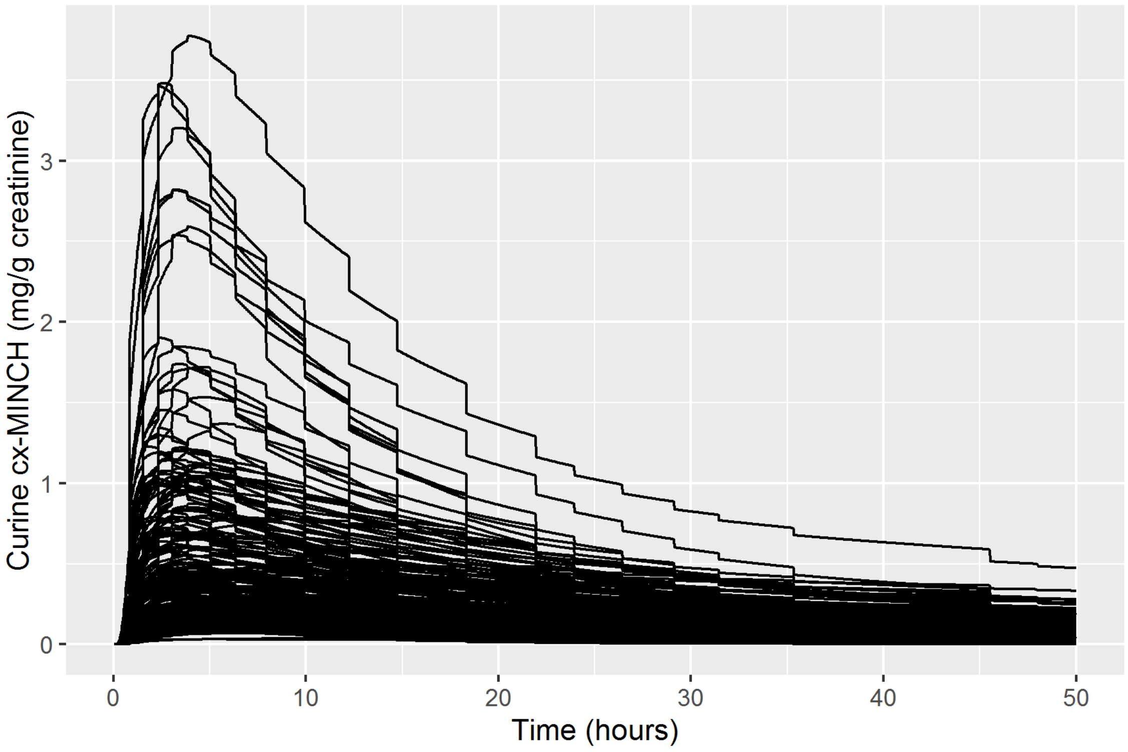

Figure S4: A comparison of 200 concentration-response profiles for the concentration of metabolite cx-MINCH expressed relative to creatinine concentration in urine

Table S1: Morris test results for CV DINCH at 0.5 and 2 hours post ingestion

Parameter	Label	0.5 hours		2 hours	
BW0	X1	0.002229	0.001562	0.001398	0.001823
VT	X2	0.015122	0.014101	0.003497	0.002482
VfaC	X3	0.008291	0.003973	0.001868	0.001443
VguC	X4	0.017078	0.011335	0.000536	0.000538
VstC	X5	0.000519	0.000669	0.000899	0.001041
VliC	X6	0.012374	0.014863	0.003248	0.00212
VspdC	X7	0.030784	0.030436	0.003366	0.002058
VrpdC	X8	0.004934	0.004063	0.000618	0.000404
VlymphC	X9	0.002001	0.001978	0.003976	0.003888
VBldC	X10	0.001327	0.001034	0.000457	0.00021
FBDINCH	X11	2.05E-06	9.56E-07	3.16E-07	5.68E-07
FBMINCH	X12	1.91E-15	3.82E-15	1.14E-13	2.18E-13
QhepartC	X13	0.000916	0.001079	0.000885	0.000971
QfaC	X14	0.005269	0.003438	0.002226	0.000804
QguC	X15	0.0151	0.013958	0.004382	0.002943
QstC	X16	0.001983	0.001668	0.000389	0.000592
QspdC	X17	0.010439	0.011609	0.00121	0.001487
QrpdC	X18	0.00797	0.003123	0.00243	0.003616
QlymphC	X19	0.002066	0.002695	0.003899	0.00505
FracMetabOH	X20	1.16E-12	1.71E-12	3.28E-09	5.20E-09
FracMetabcx	X21	4.21E-14	5.62E-14	4.30E-11	4.83E-11
FracDOSE	X22	0.038122	0.017793	0.013737	0.019606
DRINKTIME	X23	0.068832	0.076009	0.02344	0.01503
BELLYPERM	X24	0.000398	0.000646	0.000922	0.001039
GIPERM	X25	0.003797	0.006061	0.000297	0.000439
BELLYPERMlymph	X26	1.61E-05	1.86E-05	1.63E-06	1.64E-06
GIPERMlymph	X27	0.000106	9.95E-05	1.23E-05	1.23E-05
KEMAX	X28	0.007457	0.005416	0.000689	0.00067
KEMIN	X29	0.000145	0.000229	3.62E-05	7.55E-05
KAMINCH	X30	0	0	0	0
Pbab	X31	0.023999	0.036662	0.010394	0.006564
Pspdb	X32	0.001242	0.000846	0.003465	0.001785
Plib	X33	0.003967	0.003638	0.000233	0.00025
Prpdb	X34	0.00491	0.003978	0.000326	0.000423
Pfab	X35	0.002556	0.001993	0.00488	0.003816
Pstb	X36	0.001175	0.001311	0.000424	0.000615
Pgub	X37	0.026779	0.017272	0.003347	0.002849
PbaM	X38	3.90E-12	8.06E-12	4.33E-10	4.59E-10
PspdM	X39	2.97E-14	5.91E-14	2.06E-10	3.23E-10
PliM	X40	1.35E-10	2.51E-10	2.16E-09	1.24E-09
PrpdM	X41	5.89E-14	1.03E-13	7.48E-11	1.09E-10
PfaM	X42	1.10E-15	1.66E-15	4.49E-11	6.68E-11
PstM	X43	0	0	0	0
PguM	X44	0	0	0	0
MPY	X45	0.0162	0.011265	0.005516	0.002442
Dinchhalf_life	X46	0.018946	0.020064	0.008927	0.009406
Minchhalf_life	X47	6.64E-11	1.26E-10	1.35E-09	1.51E-09
MPYgu	X48	0.004269	0.006037	0.005953	0.005175
DinchGUT_half_life	X49	0.005769	0.006961	0.004326	0.0026

K1_OH	X50	3.04E-14	4.24E-14	7.45E-10	1.42E-09
K1_cx	X51	2.30E-14	4.74E-14	1.35E-10	2.59E-10
Rurine	X52	4.02E-13	5.46E-13	7.99E-11	1.23E-10
Creat	X53	0	0	0	0

Table S2: Morris test results for CV MINCH at 1 and 3 hours post ingestion

Parameter	Label	1 hours		3 hours	
BW0	X1	0.001262	0.001427	0.000514	0.000657
VT	X2	0.006239	0.006513	0.002372	0.00174
VfaC	X3	0.003865	0.003355	0.001116	0.000836
VguC	X4	0.002997	0.003487	0.000761	0.000819
VstC	X5	0.001744	0.001995	0.000593	0.000853
VliC	X6	0.010296	0.011178	0.005074	0.005225
VspdC	X7	0.013685	0.019743	0.002503	0.001197
VrpdC	X8	0.00121	0.001287	0.000623	0.000705
VlymphC	X9	0.000194	0.000174	0.000859	0.000568
VBldC	X10	0.000601	0.000594	0.000279	0.000162
FBDINCH	X11	1.73E-07	1.34E-07	4.76E-08	8.23E-08
FBMINCH	X12	4.04E-07	6.55E-07	5.37E-08	4.89E-08
QhepartC	X13	0.001874	0.002108	0.000454	0.000749
QfaC	X14	0.00268	0.002949	0.001948	0.002114
QguC	X15	0.009326	0.012058	0.000389	0.000384
QstC	X16	0.001024	0.000854	0.000349	0.000311
QspdC	X17	0.00662	0.010104	0.000677	0.000951
QrpdC	X18	0.005705	0.003748	0.001345	0.001579
QlymphC	X19	0.000166	0.000222	0.000757	0.001028
FracMetabOH	X20	1.69E-09	3.28E-09	1.07E-08	1.22E-08
FracMetabcx	X21	1.02E-10	1.56E-10	9.00E-11	1.05E-10
FracDOSE	X22	0.018241	0.012881	0.008055	0.005342
DRINKTIME	X23	0.040528	0.063195	0.016432	0.015269
BELLYPERM	X24	0.000824	0.000564	0.000131	0.000159
GIPERM	X25	0.000187	0.000259	5.59E-05	8.00E-05
BELLYPERMlymph	X26	2.09E-07	2.15E-07	3.07E-07	4.13E-07
GIPERMlymph	X27	8.26E-06	8.32E-06	2.86E-06	2.52E-06
KEMAX	X28	0.001479	0.001721	0.000118	0.00015
KEMIN	X29	6.98E-06	8.55E-06	5.89E-06	1.16E-05
KAMINCH	X30	0	0	0	0
Pbab	X31	0.005655	0.007108	0.000419	0.000404
Pspdb	X32	0.00011	0.000145	0.000505	0.000311
Plib	X33	0.000526	0.000588	3.58E-05	4.41E-05
Prpdb	X34	0.000239	0.00039	2.94E-05	3.81E-05
Pfab	X35	0.000139	0.000181	0.000629	0.000579
Pstb	X36	0.000505	0.000711	0.000114	0.000165
Pgub	X37	0.007713	0.005777	0.001252	0.001026
PbaM	X38	0.002277	0.00377	0.001697	0.001262
PspdM	X39	0.001266	0.001059	0.004018	0.002191
PliM	X40	0.00297	0.004555	0.000384	0.000532
PrpdM	X41	0.002582	0.004015	0.000724	0.000743
PfaM	X42	0.000108	8.88E-05	0.000615	0.000632
PstM	X43	0	0	0	0
PguM	X44	0	0	0	0
MPY	X45	0.006068	0.005513	0.007622	0.006339
Dinchhalf_life	X46	0.003802	0.004106	0.000621	0.000666
Minchhalf_life	X47	0.0193	0.031565	0.010109	0.008322
MPYgu	X48	0.005093	0.006448	0.000343	0.000439
DinchGUT_half_life	X49	0.004334	0.003279	0.000168	0.000229

K1_OH	X50	9.00E-12	1.07E-11	1.04E-09	1.47E-09
K1_cx	X51	6.98E-11	1.53E-10	3.73E-10	5.30E-10
Rurine	X52	7.70E-12	1.09E-11	1.25E-10	1.88E-10
Creat	X53	0	0	0	0

Table S3: Morris test results for Curine OH-MINCH at 2 and 10 hours post ingestion

Parameter	Label	2 hours		10 hours	
BW0	X1	0.264718	0.079269	0.350282	0.217625
VT	X2	0.066722	0.10097	0.014637	0.01633
VfaC	X3	0.009373	0.012265	0.017034	0.022063
VguC	X4	0.048784	0.063603	0.004739	0.006257
VstC	X5	0.014222	0.007398	0.010063	0.009232
VliC	X6	0.172346	0.292024	0.124934	0.192381
VspdC	X7	0.174746	0.258875	0.167066	0.197313
VrpdC	X8	0.004666	0.007066	0.009748	0.012794
VlymphC	X9	0.010174	0.014503	0.093451	0.090266
VBldC	X10	0.002085	0.002668	0.002721	0.00396
FBDINCH	X11	3.18E-06	9.45E-07	4.20E-06	7.30E-06
FBMINCH	X12	4.43E-06	6.37E-06	1.91E-06	2.36E-06
QhepartC	X13	0.019183	0.026774	0.007227	0.006196
QfaC	X14	0.01261	0.008597	0.042504	0.042695
QguC	X15	0.068896	0.051677	0.014458	0.017335
QstC	X16	0.018602	0.022765	0.008042	0.010535
QspdC	X17	0.025088	0.038276	0.014939	0.012926
QrpdC	X18	0.058458	0.081914	0.031551	0.044333
QlymphC	X19	0.002999	0.004522	0.042188	0.067879
FracMetabOH	X20	1.03E-07	2.12E-07	2.85E-05	4.31E-05
FracMetabcx	X21	1.002266	0.77747	0.694809	0.531791
FracDOSE	X22	0.676626	0.989185	0.348198	0.212228
DRINKTIME	X23	1.117216	1.833853	0.720769	0.736439
BELLYPERM	X24	0.028875	0.024146	0.005183	0.005491
GIPERM	X25	0.01411	0.018228	0.001538	0.001846
BELLYPERMlymph	X26	8.05E-06	8.93E-06	1.55E-05	1.54E-05
GIPERMlymph	X27	0.000425	0.000672	0.000278	0.000318
KEMAX	X28	0.031451	0.01975	0.002874	0.004188
KEMIN	X29	0.002031	0.003874	0.000254	0.000479
KAMINCH	X30	0	0	0	0
Pbab	X31	0.200739	0.276669	0.0353	0.056546
Pspdb	X32	0.004013	0.004014	0.033786	0.04677
Plib	X33	0.007612	0.006867	0.000781	0.001169
Prpdb	X34	0.005554	0.007023	0.002641	0.003558
Pfab	X35	0.014679	0.019093	0.186846	0.2054
Pstb	X36	0.013939	0.011493	0.008836	0.010332
Pgub	X37	0.113149	0.080671	0.015684	0.011849
PbaM	X38	0.103798	0.084724	0.067482	0.092521
PspdM	X39	0.001165	0.00117	0.030176	0.028544
PliM	X40	0.152183	0.142412	0.014839	0.019443
PrpdM	X41	0.008157	0.010573	0.011638	0.006469
PfaM	X42	0.00085	0.001164	0.014765	0.012325
PstM	X43	0	0	0	0
PguM	X44	0	0	0	0
MPY	X45	0.536828	0.260417	0.192023	0.168694
Dinchhalf_life	X46	0.169317	0.192511	0.06218	0.054328
Minchhalf_life	X47	0.783459	1.00019	0.208868	0.24991
MPYgu	X48	0.213546	0.293378	0.010461	0.0067
DinchGUT_half_life	X49	0.148575	0.218066	0.01075	0.014942

K1_OH	X50	1.11E-09	1.47E-09	8.39E-07	8.20E-07
K1_cx	X51	1.125815	1.90084	0.120232	0.157338
Rurine	X52	0.481621	0.335221	0.605852	0.49565
Creat	X53	0.773485	0.78323	0.796008	0.8062

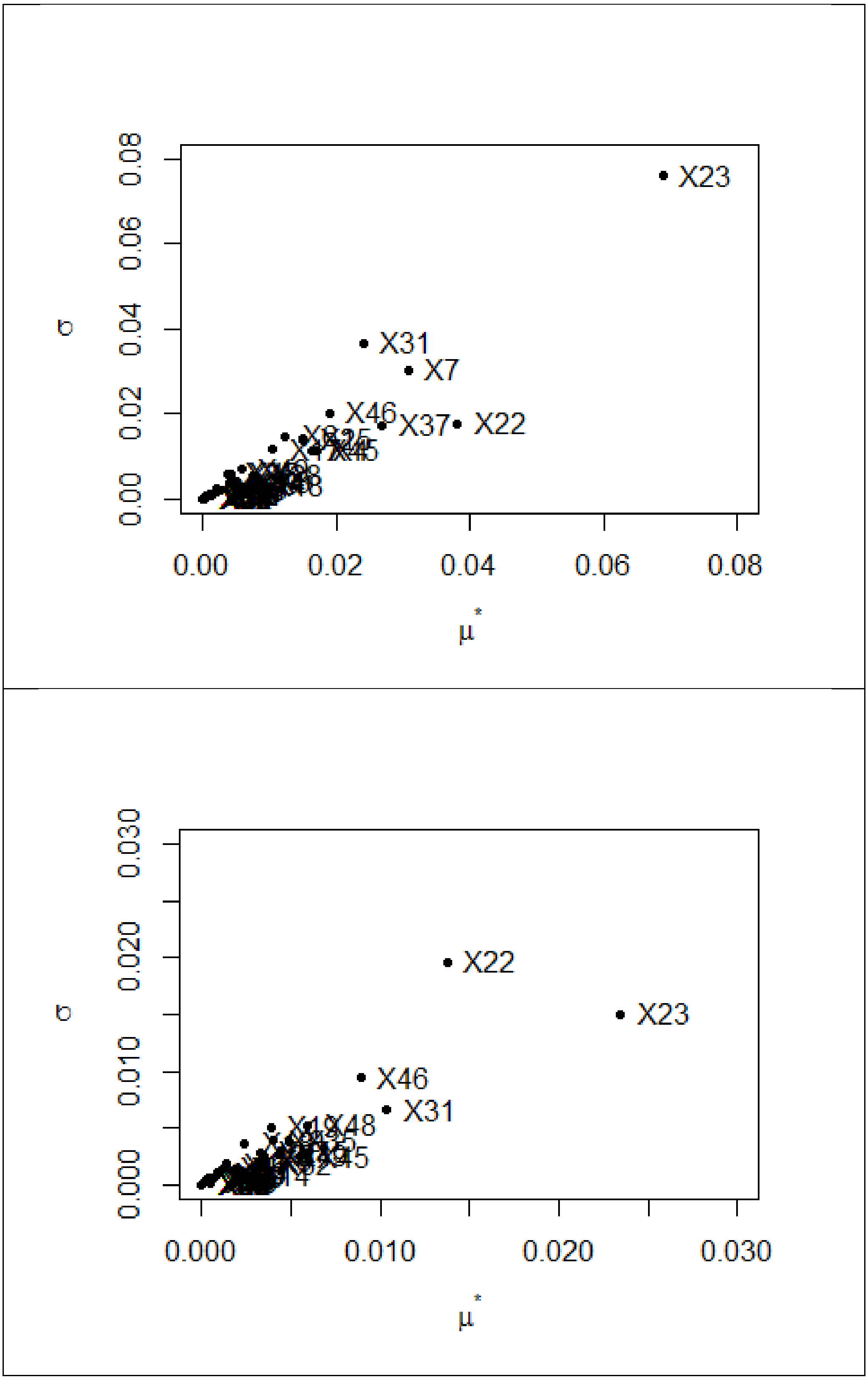

Figure S5: Morris test mu* and sigma calculated for the model output of CV DINCH at: a) 0.5 hours; b) 2 hours post exposure

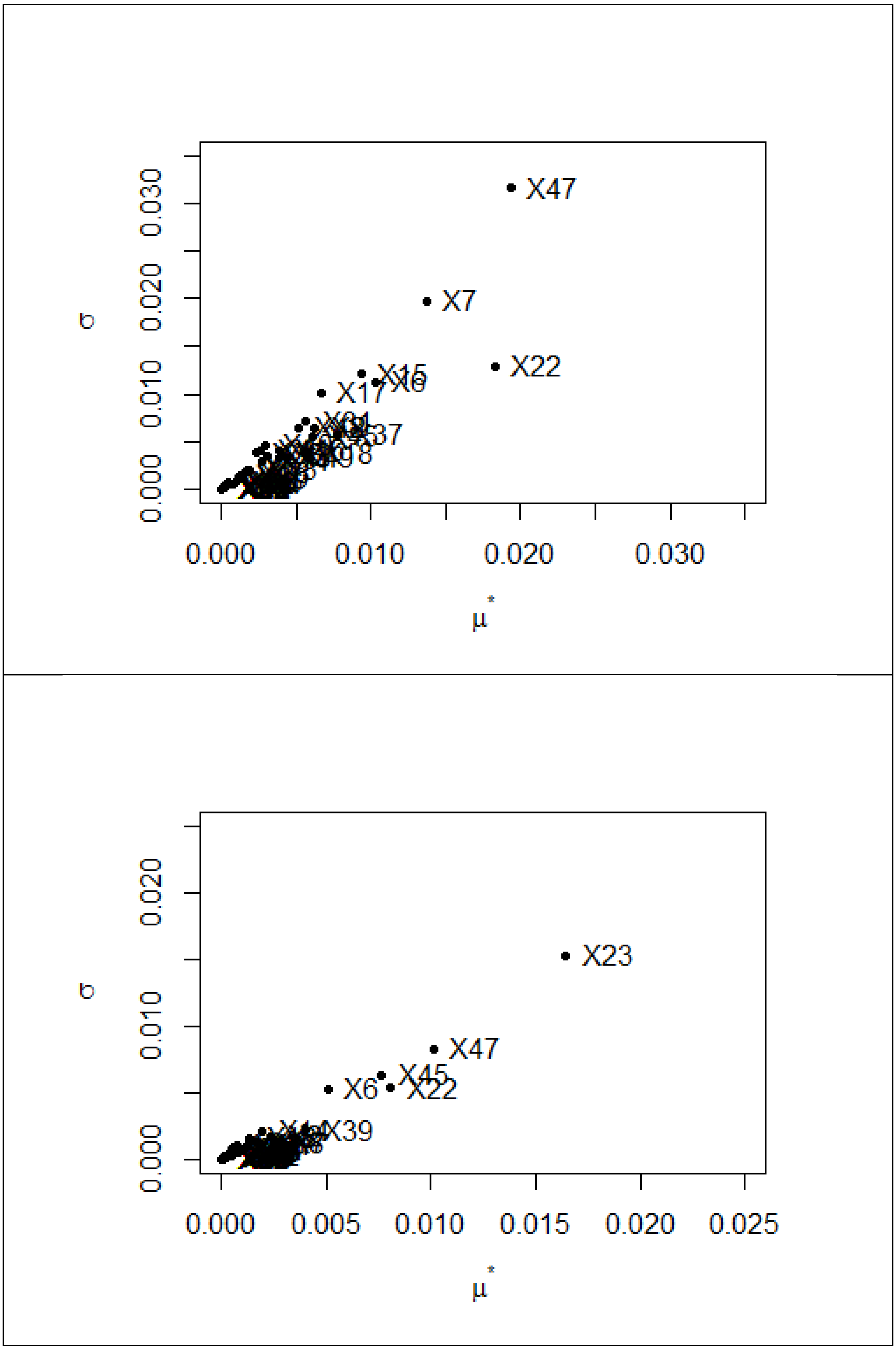

Figure S6: Morris test mu* and sigma calculated for the model output of CV MINCH at: a) 1.0 hours; b) 5 hours post exposure

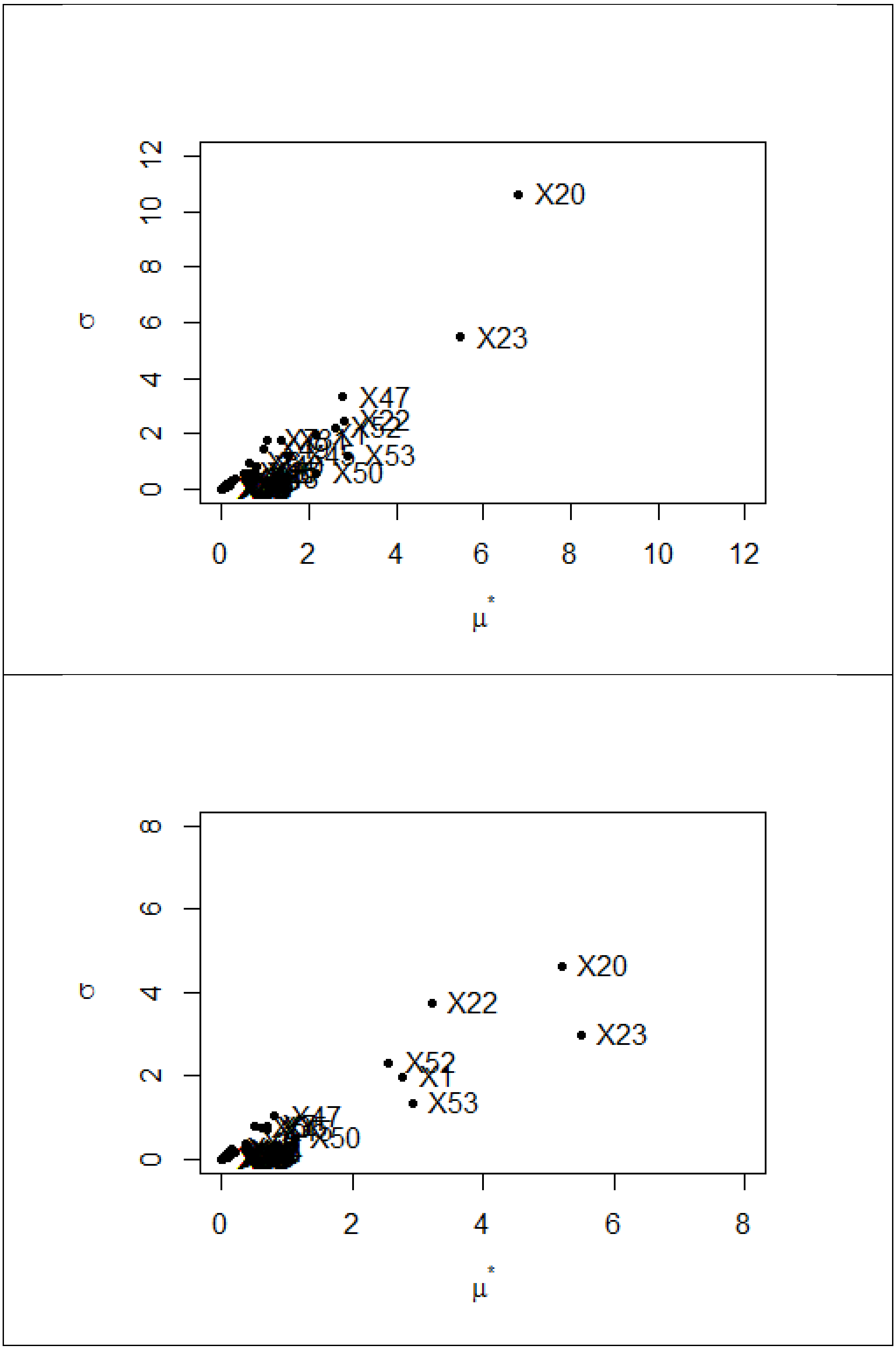

Figure S7: Morris test mu* and sigma calculated for the model output of Curine OH-MINCH at: a) 2 hours; b) 10 hours post exposure

Table 4 Tissue dosimetry in an 85 kg male

Daily Intake	Peak Conc (mg/L)	Average Conc (mg/L)	Peak Conc (mg/L)	Average Conc (mg/L)	Peak Conc (mg/L)	Average Conc (mg/L)
(mg/kg/day)	Plasma		Liver		Adipose	
0.0001	1.75E-04	4.15E-05	1.07E-03	4.24E-05	1.50E-05	1.24E-05
0.001	1.74E-03	4.15E-04	1.07E-02	4.24E-04	1.50E-04	1.24E-04
0.01	1.74E-02	4.15E-03	1.07E-01	4.24E-03	1.50E-03	1.24E-03
0.1	1.74E-01	4.15E-02	1.07E+00	4.24E-02	1.50E-02	1.24E-02
1.0	1.74E+00	4.15E-01	1.06E+01	4.24E-01	1.50E-01	1.24E-01
50	8.47E+01	2.06E+01	4.98E+02	2.11E+01	7.46E+00	6.12E+00
250	3.30E+02	9.76E+01	1.69E+03	1.00E+02	3.53E+01	2.87E+01
750	6.54E+02	2.65E+02	2.82E+03	2.74E+02	9.60E+01	7.66E+01

Table 5 Tissue dosimetry in a 70 kg non-pregnant female

Daily Intake	Peak Conc (mg/L)	Average Conc (mg/L)	Peak Conc (mg/L)	Average Conc (mg/L)	Peak Conc (mg/L)	Average Conc (mg/L)
(mg/kg/day)	Plasma		Liver		Adipose	
0.0001	2.77E-04	4.66E-05	1.09E-03	4.54E-05	1.75E-05	1.43E-05
0.001	2.77E-03	4.66E-04	1.09E-02	4.54E-04	1.75E-04	1.43E-04
0.01	2.77E-02	4.66E-03	1.09E-01	4.54E-03	1.75E-03	1.43E-03
0.1	2.77E-01	4.66E-02	1.09E+00	4.54E-02	1.75E-02	1.43E-02
1.0	2.77E+00	4.66E-01	1.08E+01	4.54E-01	1.75E-01	1.43E-01
50	1.34E+02	2.31E+01	5.09E+02	2.26E+01	8.69E+00	7.10E+00
250	5.07E+02	1.10E+02	1.73E+03	1.08E+02	4.12E+01	3.34E+01
750	9.39E+02	3.02E+02	2.90E+03	2.96E+02	1.12E+02	8.97E+01
